# Quantitative influence of macromolecular crowding on gene regulation kinetics

**DOI:** 10.1093/nar/gkt907

**Published:** 2013-10-08

**Authors:** Marcin Tabaka, Tomasz Kalwarczyk, Robert Hołyst

**Affiliations:** Institute of Physical Chemistry, Polish Academy of Sciences, 44/52 Kasprzaka, 01-224 Warsaw, Poland

## Abstract

We introduce macromolecular crowding quantitatively into the model for kinetics of gene regulation in *Escherichia coli*. We analyse and compute the specific-site searching time for 180 known transcription factors (TFs) regulating 1300 operons. The time is between 160 s (e.g. for SoxS M_w_ = 12.91 kDa) and 1550 s (e.g. for PepA_6_ of M_w_ = 329.28 kDa). Diffusion coefficients for one-dimensional sliding are between 
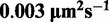
 for large proteins up to 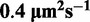 for small monomers or dimers. Three-dimensional diffusion coefficients in the cytoplasm are 2 orders of magnitude larger than 1D sliding coefficients, nevertheless the sliding enhances the binding rates of TF to specific sites by 1–2 orders of magnitude. The latter effect is due to ubiquitous non-specific binding. We compare the model to experimental data for LacI repressor and find that non-specific binding of the protein to DNA is activation- and not diffusion-limited. We show that the target location rate by LacI repressor is optimized with respect to microscopic rate constant for association to non-specific sites on DNA. We analyse the effect of oligomerization of TFs and DNA looping effects on searching kinetics. We show that optimal searching strategy depends on TF abundance.

## INTRODUCTION

For many decades, macromolecular crowding has been well advertised as a factor strongly affecting all biochemical reactions and other processes in living cells (1–4). Yet, rarely this effect has been quantitatively analysed. We follow classical works on facilitated target location (5–11), extended by Li *et al.* (12) to analyse gene regulations in *Escherichia coli* by transcription factors (TFs). We explicitly include new factors to the model: reduction of mobility of TFs in the cytoplasm and on DNA due to macromolecular crowding (13,14) and reduction of nucleoid volume caused by nucleoid-associated proteins (NAPs) covering DNA. We use the model of TF diffusion along DNA (sliding) of Blainey *et al.* (15,16). We calibrate the model using non-specific binding constant of LacI to DNA calculated from single-molecule experimental data (17,18). Next we apply the model to all known 180 TFs that bind specifically to *cis*-regulatory elements and regulate 1300 operons. In particular we determine 3D and 1D diffusion constants for TFs, enhancement rates due to the sliding and times for location of operators by TFs. We discuss various strategies used by TFs for rapid target location *in vivo*. We provide a large number of data, which can be used for analysis of genetic networks in systems biology. These data can also guide future experiments, since quantitative experimental data *in vivo* are available for only one repressor (LacI) regulating only one operon.

**Figure 1.**
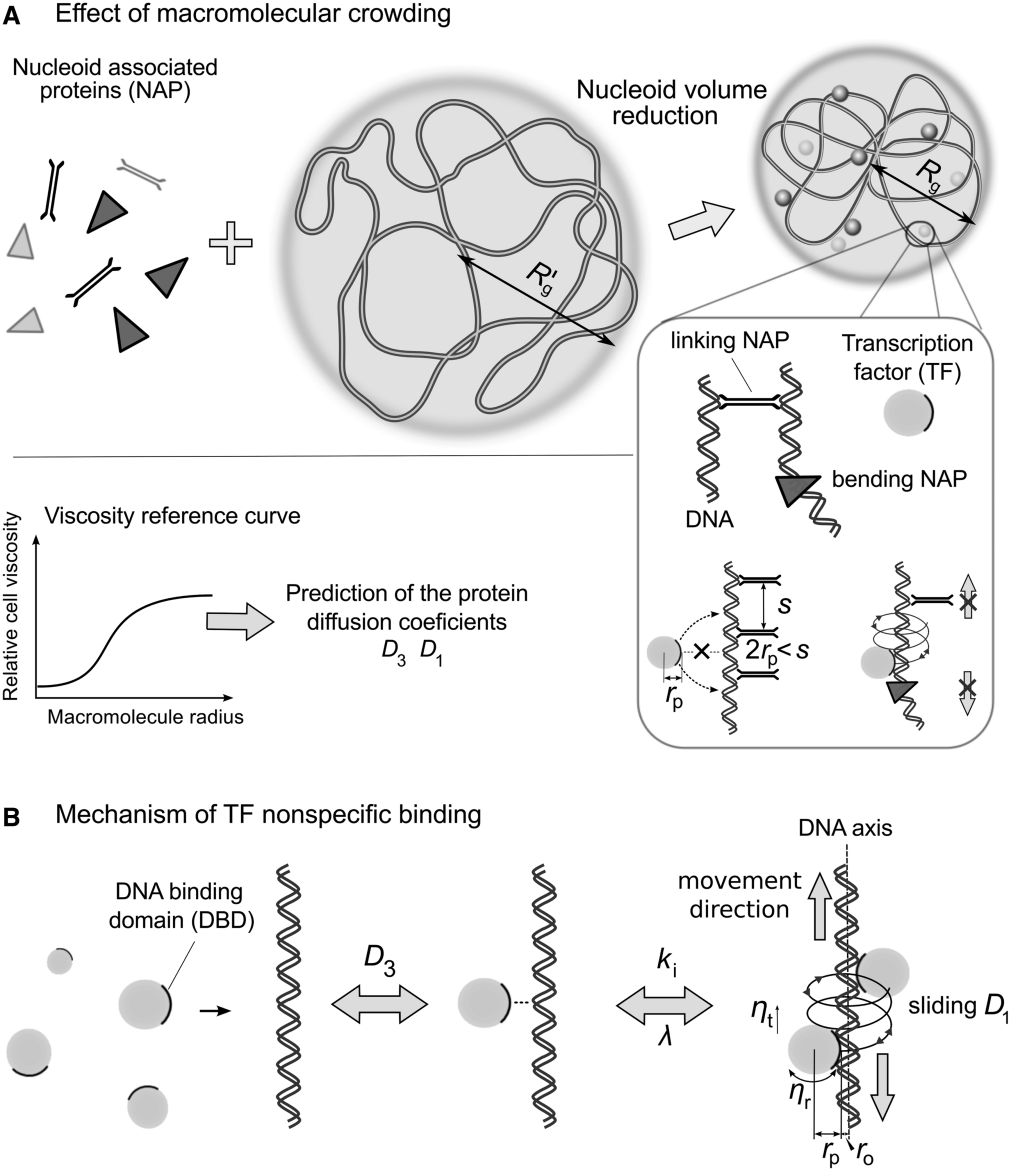
Influence of macromolecular crowding on gene regulation kinetics. (**A**) NAPs cause shrinking of available searching volume by linking and bending of DNA chains. NAPs when bound to DNA disturb non-specific association of TFs and 1D diffusion along DNA (12). Macromolecular crowding increases viscosity of the cytoplasm and diminishes TF 1D and 3D-mobility constants [Equations (1–2)]. The viscosity experienced by a translocating macromolecule is a stretched exponential function of size of the macromolecule (13,14). (**B**) TF searches for specific site on DNA through numerous DNA–non-specific associations. Three-dimensional diffusion brings TF close to DNA. Next, TF binds to DNA with microscopic association rate constant *k_i_* (8). Non-specifically bound TF slides performing curvilinear motion (16,19) along DNA and dissociates with rate constant λ.

TFs use diffusion driven by thermal fluctuations to search for specific sites on DNA. The search process combines 3D diffusion of TF through a cytoplasm, subsequent non-specific binding of TF to DNA, next 1D diffusion along a DNA and finally unbinding into cytoplasm (and subsequent repeat of the previous steps) or target recognition when TF encounters an operator during sliding (Figure 1). This process is known as the facilitated target location (5–11). Typical bacterial genome consists of 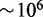 base pairs, and location of the specific site on DNA is through enormous amount of bindings to non-specific sites. One-dimensional diffusion along DNA increases effective target size on DNA and speeds the searching up to 2 orders of magnitude (8,12,18). Recent *in vivo* single-molecule experiment has shown that LacI repressor dimer needs 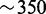 s to locate the operator (17) and on average slides along 36 bp when non-specifically bound (18) to DNA. The diffusion coefficient for sliding has been measured in the buffers only (16,17).

The prokaryotic cytoplasm is a highly crowded environment (14,20–22). Two aspects of macromolecular crowding affect regulation of gene expression (Figure 1A): (i) cytoplasmic crowding reduces 3D and 1D diffusion constant of TF and (ii) crowding on DNA disturbs TF non-specific binding and 1D diffusion along DNA due to presence of NAPs (12,23,24). However, NAPs additionally shrink the nucleoid, thereby decrease the size of space where the process of facilitated target location occurs. Elowitz *et al.* (25) have shown that protein diffusion in the *E. coli* cytoplasm is the normal diffusion, but significantly reduced as compared with its mobility in water. This reduction is a non-linear function of protein molecular weight (21,22). We have introduced the phenomenological model of cytoplasm length-scale dependent viscosity (13,14) to describe all available experimental data for diffusion of small ligands, proteins and DNA plasmids in *E. coli*. We have previously tested the model (13) for motion of proteins in buffers crowded by polymers (13,26) or micelles (27). In this article, we apply the model to describe the influence of macromolecular crowding on 1D diffusion along DNA. There is a marked difference between diffusion in the cytoplasm and sliding. During sliding on DNA, the protein follows the path along major grooves and thus rotates around its own axis of rotation and also around the DNA. The friction for rotational motion is proportional to the volume of the protein (15,16), whereas for the translational diffusion in the cytoplasm, the friction is proportional to the radius of the protein. One-dimensional diffusion coefficients are substantially (2 orders of magnitude) smaller than 3D diffusion constants for the typical sizes of TFs.

## MATERIALS AND METHODS

### Macromolecular crowding

The prokaryotic cell is a densely packed structure with concentration of macromolecules between 200 and 400 g/l (20–30% of cellular volume) (20,28). This macromolecular crowding in the cytoplasm reduces in the first place diffusional mobilities of TFs. The reduction of mobility is proportional to the scale-dependent viscosity (13,26). This viscosity describes the mobility in complex liquids (13,26), in cytoplasm of the eukaryotic cells (HeLa and Swiss 3T3 fibroblasts) (13) and prokaryotic cells (*E. coli*) (14). This model based on the viscosity describes protein–protein association kinetics in Hela cytoplasm (29). The model is based on the Stokes-Sutherland-Einstein relation 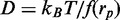, where *D* is a diffusion constant of macromolecule, 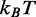 is the Boltzmann constant times the absolute temperature and *f* is the length-scale–dependent hydrodynamic drag friction. For 3D diffusion coefficient *D*_3_, this drag has the following form:
(1)
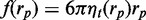



Thus 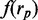 is proportional to the length-scale–dependent viscosity 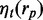. At fixed temperature, the viscosity 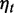 experienced by an object of hydrodynamic radius *r_p_* is described by the formula (13,26) 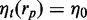 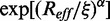, where 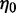 is the solvent viscosity, ξ is the correlation length, 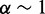 is a constant and effective radius *R_eff_* is defined as 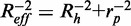, where *R_h_* is the effective hydrodynamic radius of macromolecular crowding agents. We find for *E. coli* these characteristic parameters by fit to experimental data: 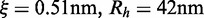 and 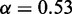 (14). The length-scale–dependent viscosity of *E. coli* cytoplasm change by a factor of 10^4^ from 0.001 Pas for small solutes to 13 Pas for large plasmids (250 nm). The calculation of the 1D coefficient *D*_1_ is based on an observation that TFs spin while non-specifically bound to DNA (16). The friction experienced by TF of size *r_p_* during its curvilinear motion along DNA is given by the following expression (19):
(2)
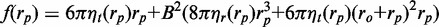

where the first term corresponds to frictional force experienced by TF bound to DNA during translational motion parallel to the DNA axis, the second term describes rotational friction that arises from TF rotation along its axis, and the last term is the rotational friction experienced by TFs that spin around DNA axis (see Figure 1B). TF makes full 360° rotation (2π rotation) around DNA after translating over the distance of 3.54 nm, i.e. 10.4 base pairs along DNA (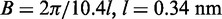 is a length between consecutive base pairs). *r_o_* accounts for the protein offset from the DNA axis (Figure 1B). We use the value of 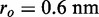 (19,30). Brownian dynamics simulations (21) have shown that rotational viscosity experienced by rotating proteins of molecular weights in the range 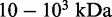 in *E. coli* cytoplasm is ∼10 times larger than water viscosity. Therefore, we assume that 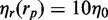.

### Nucleoid volume

NAPs reduce volume of the nucleoid (Figure 1A), by bending and linking DNA strands. There are two types of proteins belonging to NAP family that affect nucleoid volume: bending NAPs that bends DNA and change its Kuhn length and linking NAPs that connect neighbouring DNA chains. We describe the volume reduction using a model of a randomly cross-linked polymer chain of the Kuhn length κ, consisting of *N* base pairs subjected to *M_l_* uncorrelated cross-linking constraints (31). The radius of gyration *R_g_* of the polymer is (31):
(3)
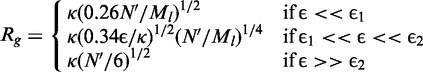

where *ε* is a mean distance between monomers that form a cross-link, 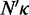 is a contour length of the polymer and 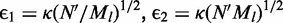. The first case in Equation (3) concerns rigid cross-linking polymer regime where 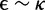 or smaller, second soft cross-linking regime and the last one describes free polymer chain regime. The volume occupied by nucleoid is calculated from 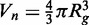. The mean distance between bending proteins is 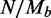, where *M_b_* is a number of bending NAPs. The Kuhn length is a non-linear function of the mean distance between bending proteins (32,33).

### Model of facilitated target location

We follow the classical works of Berg *et al.* (6–8) with the extension by Li *et al.* (12). The TF association rate constant to a specific site on DNA is as follows:
(4)
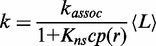



The association rate constant is equal to the rate constant per binding site *k_assoc_* diminished by the presence of non-specific sites on DNA. *K_ns_* is the equilibrium constant for non-specific binding of TF to DNA, *c* is the concentration of non-specific DNA, *p*(*r*) is the probability distribution that the binding site is free of NAPs and *r* is the diameter of TF.
(5)
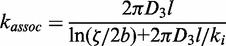

where *k_i_* is a microscopic (intrinsic) association rate constant (Figure 1B), *l* is a distance between consecutive base pairs and ζ is a mean distance between neighbouring DNA chains and *b* is the DNA radius. ζ is calculated as in (8,12) from 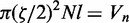. The probability that a binding site of size *r* on DNA is free from NAPs is as follows (12,24):
(6)
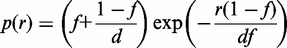

where *d* is a mean weighted size of NAPs, i.e. 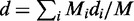, with *M* being the total amount of NAPs per nucleoid and 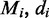 are amount and size of *i*-th NAP (see Supplementary Table S1), respectively. The fraction of DNA that is free from NAPs is 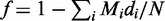. The sliding enhances the rate constant by effectively increasing the mean target size 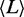. The size (in bp), under the assumption of semi-stationary positions of NAPs (12), is as follows:
(7)
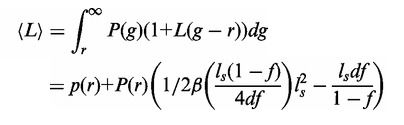

where *P*(*g*) is the probability density function calculated from Equation (6), 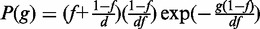, and 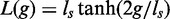 is an effective target size for specific site placed symmetrically between two NAPs (8,12) with 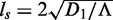. *β* is the 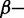 function (34) given by 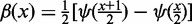 with ψ being an Euler psi function (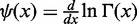). The factor 1 denotes that at least one binding site is scanned on TF binding. Λ is the macroscopic dissociation constant (dissociation to the distance 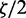 where TF can bind to uncorrelated DNA chain) equal to microscopic dissociation constant λ times the probability of reaching 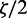 (8), that is
(8)
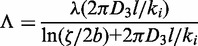



## RESULTS

### The presence of NAPs highly reduces nucleoid volume

The length of *E. coli* nucleoid is 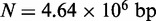. The Kuhn length of undisturbed DNA is 300 bp, 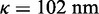. For the free polymer regime (without NAPs bound to DNA), the nucleoid radius of gyration and volume would be 
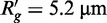
 and 
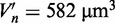
, respectively, that is much larger than *E. coli* cell size. Abundances of DNA binding proteins [NAPs (35,36), TF (37) and RNA polymerase (RNAP) (38)] for early exponential phase of *E. coli* growth in rich media are collected in Supplementary Table S1. The calculated fraction of DNA that is free from DNA binding proteins is 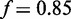 (total level of the proteins is *M* = 36 600). Number of bending NAPs 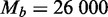 shows that mean distance between these NAPs (178 bp) is below Kuhn length of naked DNA. The random placement of bending protein every 200 bp reduce the persistence length by a factor 2.2 (39). We use the value 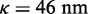 in calculations. Thus, we predict that nucleoid shrinking is due to presence of DNA linking and bending proteins. The observed volume of nucleoid is within range 1–0.1 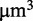 (40–43). In Supplementary Figure S1, we show that the number of cross-links estimated from (44,45) is enough to shrink nucleoid to the observed values. The length of relaxed MukB homodimer is 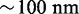, while on binding to DNA, the protein is compacted to the size below the Kuhn length of DNA (46). Similar case is for SeqA and MatP proteins (47). This is significantly below calculated value of 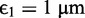. Therefore, the nucleoid is in the first regime of Equation (3). The mean volume of the nucleoid is between 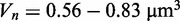 at the early exponential phase of growth (Supplementary text and Figure S1). Two other NAPs are commonly attributed to the group of NAPs forming connections between DNA chains: H-NS/StpA dimers (48,49) and Dps dodecamers through the oligomerization (50). However, presence of divalent cations (Mg^2+^) precludes formation of bridges between DNA chains by these proteins (50,51).

### Non-specific binding of LacI to DNA is activation-limited

We use the results of single-molecule experimental studies (17,18) of specific-site binding kinetics of LacI dimer (fused with Venus proteins). We obtain parameters for the model in the following fitting procedure: (i) In Ref. (18), the fit giving sliding length 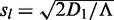 does not include presence of NAPs. We develop a simulation procedure (Supplementary text and Supplementary Figure S2) for this experiment, including NAPs on DNA as crowders. The best fit to the model gives 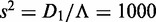 bp^2^ that corresponds to sliding length equal to 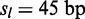 (for the vacancy 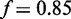, and mean weighted size of NAPs *d* = 19 bp). The sliding length is larger in our model since NAPs weaken the spatial correlations between operators. (ii) The 3D diffusion constant of LacI dimer without DNA binding domains equals (17) 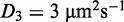. We use this value for calculation of hydrodynamic radius of LacI dimer fused with Venus proteins from Equation (1). We get 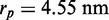. The calculated 1D diffusion constant for *in vivo* conditions is 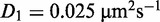. (iii) From point (i) and (ii), we calculate 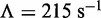. (iv) The non-specific equilibrium constant *K_ns_* is calculated from the equation 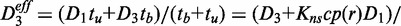 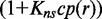, where *t_b_* and *t_u_* are the average times spent by the protein diffusing in the cytoplasm (with diffusion constant *D*_3_) and spent diffusing on DNA (with diffusion constant *D*_1_), respectively. The concentration *c* of non-specific sites is 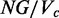, where *G* is the amount of DNA in genome equivalents and *V_c_* is the cell volume (Supplementary text). For 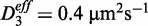 (17), we get the value 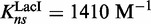. (v) From Equation (8), we calculate microscopic parameters: 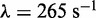 and 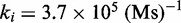. The 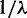 gives the average time spent by the protein on DNA smaller than 5 ms as observed *in vivo* (17). We get 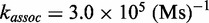. Such small value of *k_assoc_* suggests that the process of binding of the protein to DNA is activation-limited since in the case of the diffusion-limited association this parameter would have the value 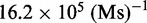. (vi) Using parameters from points (i-v) we find from Equations (4–8) that searching of the single specific site takes 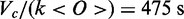 for LacI dimer fused with Venus proteins, where 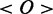 is the average number of operators in the cell volume (Supplementary text). The lower value of *s*^2^ in the fit from pt. (i) is 
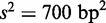
 and this gives the time 385 s. The experimentally determined rate is 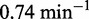 and there are 3–5 LacI dimers (18). Thus, the *in vivo* binding time is in the range (240–405 s).

### LacI repressor and RNA polymerase are optimized for fast target location

The average time for target location is a function of microscopic association and dissociation constants and can be reduced by changing these two constants. In the previous paragraph, we have discussed the experiment involving LacI dimer, but LacI protein in the native form attaches to the operator as the tetramer. Molecular weight of LacI tetramer is 154.36 kDa. Its hydrodynamic radius (in nm) is determined from the following equation (52):
(9)
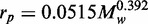



**Figure 2.**
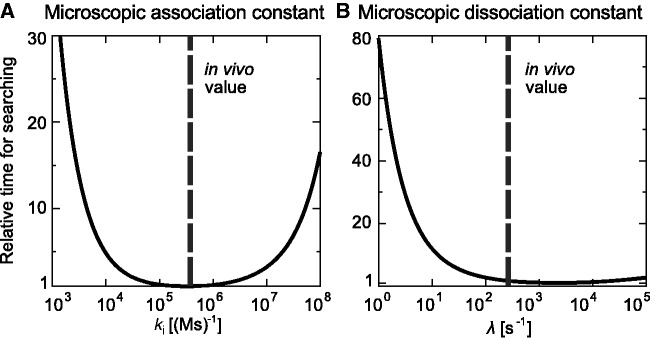
Relative changes of the average time for location of the operator by LacI tetramer. (**A**) Microscopic association constant is optimized for fast target location. The times are given with respect to the minimal value. (**B**) The minimum of the time as a function of the microscopic dissociation rate constant is shifted by 1 order of magnitude from determined value. However, the decrease of the time is only by a factor 1.66. The dashed lines point to determined values of microscopic rate constants.

The calculated mobility constants (14) 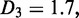 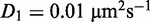 give the overall search time equal to 730 s. Figure 2 depicts the relative average time of location of the specific site as a function of one of the microscopic rate constants (the second is kept constant). We find that the searching time has a minimum as a function of microscopic association rate constant *k_i_*. The minimization is an effect of two opposite processes. Increase of microscopic constant enhances the rate of TF binding to non-specific site and increase the overall association rate [Equation (4)]. The increase is limited as *k_assoc_* becomes diffusion-limited, i.e. for 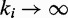. Additionally it increases non-specific equilibrium constant and decrease the overall rate by tight binding to non-specific sites. For large values of *k_i_*, TF is recaptured to the same site after dissociation, and thus the search process is limited by 1D sliding. The association constant for *in vivo* conditions differ from that in the minimum by a factor 1.002.

The reduction of association rate due to transient binding to non-specific sites [by a factor 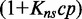, denominator of Equation (4)] is larger than decrease of the effective target length due to the sliding 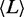. Therefore, the time for target location reaches a constant value before sliding length is reduced to 1 bp. The minimum is for 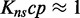 (8). It corresponds to 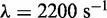 (Figure 2B), i.e. it is 1 order of magnitude larger than calculated for *in vivo* conditions. However, the minimum is shallow and the calculated difference in search time for *in vivo* conditions and at the minimum is only 66%. We suspect that high dissociation rate for optimal searching time is not possible for a large macromolecule.

We have done similar analysis for *E. coli* single promoter localization time during transcription initiation. Calculation of the mobility constants for RNA polymerase holoenzyme complex (with 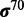 subunit bound, 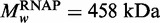) gives 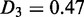 and 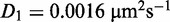. The microscopic dissociation constant, 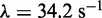, for RNAP has been recently determined (53) from single molecule measurements. The equilibrium constant for non-specific binding of RNAP to DNA has not been measured so far *in vivo*, but its estimation for cellular conditions gives a value 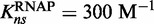 (54) and *in vitro* measurements for high ionic strength with divalent cations that mimics milieu of *E. coli* cytoplasm gives 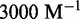 (55). In Supplementary Figure S3, we show that minimum of searching time as a function of microscopic association time is between *k_i_* values calculated from the above equilibrium constants. It has been observed recently in independent *in vitro* experiments that RNA polymerase with 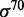 (53) or 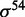 (56) factors does not perform long-range sliding when searching for its promoter. Our calculations also give short-range sliding of the length 28 bp for RNAP-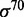 complex in the cell. The measured lifetime of RNAP-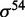 with non-specific DNA is equal to 4.7 s but such long non-specific binding was not observed for 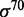 complex (53) pointing that this subunit may interact differently with non-specific DNA.

### Diffusion constants and average time for the location of a single site on DNA for all recognized TFs

**Figure 3.**
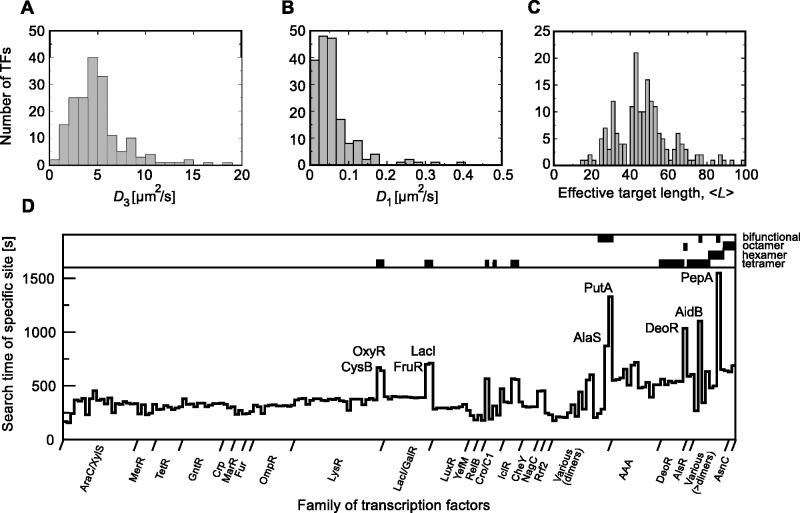
Analysis of TF mobility and kinetic parameters for the set of recognized TFs (Supplementary Table S2) from *E. coli* EcoCyc (58) and RegulonDB (59) databases. Histograms of TF (A) three-dimensional, *D*_3_ [Equation (1)] and (**B**) one-dimensional diffusion constants, *D*_1_ [Equation (2)]. (**C**) The histogram of the effective target enhancement factors 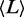 [Equation (7)]. (**D**) The average time for the location of single specific binding site by TFs [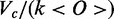]. For simplicity, we assume that 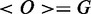. TFs are grouped into families (the order is the same as in Supplementary Table S2). In the upper belt of the panel (D), TFs that form stable higher oligomers (oligomerization level higher than 2) and/or are bifunctional (TF poses DNA binding domain and various enzymatic activity) are marked with black stripes.

The *E. coli* genome contains ∼300 genes that encode proteins that regulate promoter activity (57). So far, more than half of them have been verified experimentally. Here we analyse kinetic and dynamic parameters characterizing the process of target location for all 180 verified TFs (regulating ∼1300 operons) for *in vivo* conditions. We get a list of all TFs and information about their monomer molecular weights (Supplementary Table S2) from *E. coli* EcoCyc (58) and RegulonDB (59) databases. We extend the EcoCyc database for TF oligomerization levels by mining of this parameter from the literature (References in Supplementary Table S2). We assume oligomerization level that is most common in a given TF family, if this information is unavailable for a given TF from this family. In Figure 3A–B, we show histograms for calculated 3D and 1D diffusion constants for all TFs. The TF mobility on DNA is 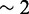 orders of magnitude slower than its cytoplasm mobility. The calculation of single-site localization times on DNA by TFs requires additional assumption about non-specific microscopic rate constants. The unknown rate parameters for all TFs (*k_i_* and λ) are assumed to be equal to that for *lac* repressor. The assumption is justified by the fact that nature of non-specific interactions of TF with DNA is primarily electrostatic (30,60,61) between the positively charged side chains of proteins and the negatively charged backbone phosphate groups of the DNA. Dahirel *et al.* (62) have shown that DNA–protein non-specific interaction free energy has a minimum at finite separation between interacting interfaces equal to 0.6 nm that is in agreement with the distance observed for the non-specific LacI–DNA complex (30). The minimum is an effect of the concave shape of DNA–proteins and an enhancement of the osmotic pressure at short separation distances. They performed also statistical analysis of 77 DNA binding proteins finding for sequence-specific DNA proteins similar interface area and mean charge density. Thus, it is expected that non-specific interaction should not vary between TFs. Moreover, *in vivo* experiments give similar values of the fraction of various non-specifically bound TFs on DNA: λ phage regulatory protein CI in λ-infected *E. coli* cells was measured to be 86% bound to non-specific sites (63) [non-specific equilibrium constants for CI and Cro repressors were identical (63)], 87% of LacI dimers fused with Venus proteins from single-molecule experiments (17) and 90% of LacI tetramers from measurements of the distribution of repressors in minicell-experiment (64).

The site-specific location times for higher-order oligomers and bifunctional TFs are substantially decreased. The association rate effective enhancement factor 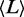 (Figure 3C) due to the sliding changes by 1 order of magnitude (between 10 for TF with large molecular weights/higher level of oligomerization to 100 for TFs that are monomers or small dimers). In Figure 3D, we present the calculated TF searching times for single specific site. The TFs are grouped in the families they belong to (Supplementary Table S2). We mark in the upper belt the TFs that form stable higher-order oligomers in solution (oligomerization level 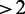) or are bifunctional, i.e. contain both DNA binding domain (DBD) and active centres responsible for enzymatic activity (e.g. AlaS - alanyl-tRNA synthetase, PutA - flavoprotein that acts as enzyme catalysing reactions of the proline degradation, AidB - isovaleryl-CoA dehydrogenase activity, PepA - aminopeptidase). Within the same family, the searching times are usually similar (except they belong to one of above groups).

### TF’s searching strategies (higher oligomers versus dimers)

A TF binding time to the regulatory region of the operon can be reduced by increasing TF concentration. Downside of this is an additional cost of protein production (65,66). The best strategy for fast regulation seems to be reached by small monomers with bipartite helix-turn-helix (HTH) motifs (as for MarA, SoxS) and single binding site in the regulatory region. However, this is not the case observed for *E. coli* regulatory motifs. Frequently, regulatory regions of the operons contain many binding sites and transcription regulation is a complex function of the number of these sites (67,68). TF dimers are the most frequent oligomeric forms. Stable higher-order oligomers occur for TFs that regulate transcription of the operons involved in transport and catabolism of sugars [e.g. lactose (LacI), glycerol-P (GlpR), glucitol (GutR), l-ascorbate (UlaR), deoxyribonucleoside (DeoR), N-acetylgalactosamine (AgaR), cell wall sugars (MurR), d-allose (AlsR)]. Higher oligomers are capable of forming DNA loops (69) that efficiently stabilize the TF–DNA complex with dissociation rates, e.g. for LacI tetramer as low as 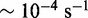 (9,70). DNA looping reduces additionally searching time for the main operator (12) within regulatory region. Many motifs are used to take advantage of DNA looping: presence of auxiliary operators within structural genes (e.g. LacI, GlpR), within regulatory regions (DeoR, AgaR, PepA, IclR), or presence of single regulatory region with multiple operators within divergent operons (AgaR, MurR, AlsR, YqjI). If auxiliary operators are separated by distances higher than sliding length of TF, they reduce searching time by a factor equal to the number of all operators (12,18). On the other side, other repressors, e.g. GalR and GalS, use DNA looping but binds to operators as dimers. Therefore, we pose a question: what is the optimal strategy for fast regulation in case when TFs use DNA looping.

**Figure 4.**
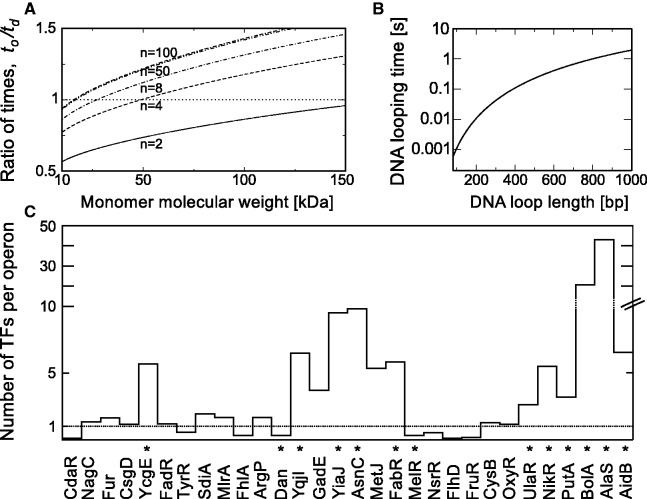
Searching strategy for the formation of TF complex at the operator depends on the number of searchers. (**A**) Ratio, 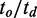, of the complex formation time via searching by oligomers (abundance *n*/*o*) to the time of independent searching by dimers (abundance *n*). Case with *o* = 2 (TF complex is a tetramer), and two independent specific sites on DNA within operator region (*m* = 2). (**B**) DNA looping times as a function of base pairs that form a loop for *in vivo* macromolecular crowding. The time calculation is based on DNA relaxation times (71,72) of size *L* from 
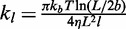
. The hydrodynamic radius of DNA is 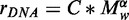 with 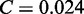 and 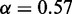 (73) and molecular weight of the 1 bp is 660 Da. The viscosity η experienced by moving DNA is calculated as previously (14). (**C**) Mean number of oligomeric TFs per regulated operon. Data are taken from single-molecule studies of protein copy number in *E. coli* cell (37). The dotted line corresponds to the ratio value equal to 1. By means of stars we marked TFs that regulate only one or two operons.

We analyse the simplest searching strategies in which regulatory region is composed of *m* independent operators and TF is a complex with oligomerization level of dimers equal to *o*. We consider two scenarios of formation of an oligomeric TF-operators complex: (i) via independent searching by *n* dimers and oligomerization of dimers when specifically bound via DNA looping (formation time 
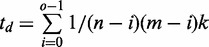
) or (ii) via searching by *n*/*o* oligomers and formation of DNA loops by means of auxiliary operators with time 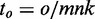. The optimal strategy depends on the number of searchers (*n* dimers versus 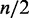 tetramers, case *m* = *o*) (Figure 4A) and molecular weight of the TF monomer. Assumption of equal number of TF monomers excludes effect of costs of TF production. Case with one stable tetramer is favoured over independent searching by two dimers. The second scenario is the best strategy for high concentration of TFs. We exclude from calculations the characteristic times of DNA loop formation, since this process for *in vivo* cellular crowding conditions is expected to take place orders of magnitude faster than specific site searching (Figure 4B).

Operons are regulated by low-copy number of TFs. We use data from single-molecule studies of protein copy number in *E. coli* cell (37) to get abundances of oligomeric TFs. We compare the values with the numbers of regulated operons [taken from RegulonDB (59)]. TFs for which both information are available are shown in Figure 4C. Divergent operons with the same operator are counted as a single unit. The results shed light on the problem of trade-off between TF’s speed of transcription regulation and costs of TF synthesis. TFs that regulate large regulons are present approximately in copies that correspond to single TF per operon. Contrary, regulons that consist of few operons (marked by stars in Figure 4) appeared to be regulated by TF levels higher than one copy per operon. In this group, the most abundant TFs are bifunctional proteins that reduce significantly searching times for specific sites. Possible explanation of these results arises from the fact that the average binding time of TF to the operator of the specific operon in a regulon decreases with the regulon size (Supplementary Figure S4). We consider the simplified problem in which the number of TFs equals the number of regulated operons and every operon contains single operator. Even regulon that consists of four operons and four TFs reduces, on average, the binding time to the particular operator by 50%. When the number of operons increases to 40, the reduction is 90%. The same effect can be achieved when TF regulates single operon by increasing the number of TFs 2- and 10-fold, respectively.

## DISCUSSION

We present a first comprehensive study of the impact of macromolecular crowding on the gene regulation kinetics in *E. coli*. Our approach combines the theory of scale-dependent diffusion (13,26), nucleoid shrinking by NAPs (31) and model of facilitated target location (8,12). This methodology is crucial for predictions of 3D and 1D diffusion constants and specific-site location times for all currently known TFs. From the parameterization of the model, we find that binding of LacI repressor to non-specific DNA is activation-limited reaction. Moreover, the microscopic association rate is optimized for fast target location for *in vivo* conditions.

From the seminal work of Riggs *et al.* (74) on association kinetics of LacI repressor to the operator site, there is broad interest in processes involved in a target location by TF (75). Recent measurements of TF searching (17,18) point that TF make use of the processes of facilitated diffusion to locate its specific site *in vivo*. Here, our analysis shows that presence of 1D diffusion speeds up the specific-site location for all TFs by 1–2 orders of magnitude as compared with the case where the TF binds with the same affinity to non-specific sites but does not slide.

The previous models concerning *in vivo* facilitated diffusion of LacI repressor (76,77) pointed that the repressor search can be close to the optimal conditions for target location. Koslover *et al.* (76) used value of LacI dissociation constant equal to 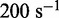 that comes from the observation that the upper value of LacI unbinding time is 5 ms (17). Both models pointed that the DNA configuration has insignificant impact on the search time. In our work, we calculated all the parameters for LacI repressor on the basis of *in vivo* data. Specifically, taking the results of experiments by Hammar *et al.* (18) and the macromolecular crowding present in the cell, we calculated the value of microscopic parameters proving that LacI optimizes the target location with respect to the binding rate constant. Thus the parameters include implicitly (78) macromolecular crowding present in the cell.

Measured 1D diffusion constant *in vitro* is 
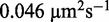
 (17) in a reaction buffer characterized by water viscosity. The value is less than predicted from Equation (2). The difference is explained by presence of small energy barrier in helical sliding of TF along DNA during transition between consecutive base pairs (16). This effect was incorporated by additional factor 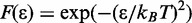 (79) multiplying right-hand side of Equation (2), where ε is root-mean-squared roughness of the interaction potential. The value of ε for LacI was determined to be 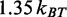 (16) for experiment carried out without Mg^2+^ in the reaction buffer. One-dimensional diffusion on DNA is much faster in the presence of Mg^2+^ (70,80). It has been shown (70) that magnesium cations change the diffusion constant of LacI repressor from 
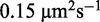
 in the presence of 10 mM Mg^2+^ to 0.045 µm^2^s^−2^ for buffers without Mg^2+^ [the divalent cations stabilize the double helix of DNA (81) and concentration of Mg^2+^ *in vivo* is evaluated to be 5–10 mM (61) or even 100 mM (82)]. More studies are required to quantify this effect as a function of Mg^2+^ concentration. We simply assume that protein 1D diffusion constant is decreased only by cytoplasm viscosity. Including energy barrier 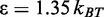 into the model gives the following values: 1D diffusion constant 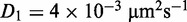, microscopic dissociation constant 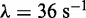 and target location time 3000 s. The values disagree with the experimental observation for which 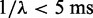 and searching times are 1 order of magnitude smaller.

TF must scan DNA base pairs (18) to recognize specific site (23,83,84). We neglected scanning in the model at non-specific sites because the scanning rates are unknown. It has been shown by Sheinman *et al.* (84) that model in which the scanning energy barrier varies between non-specific sites gives appreciable increase of searching times. The free-energy calculations between DNA and protein give argument for the existence of such barriers (62). The model allows also for optimal search that is equivalent to the classical model of facilitated target location. Since the calculated value of searching time for LacI dimer is similar to the experimentally measured one, we suspect that the TF trapping at non-specific sites does not affect significantly binding times.

We assume that motion of all TFs and RNAP is diffusive. This is confirmed by many experimental results for proteins of large range of sizes [(14,22,25) and references therein]. Genomic loci (85) and large RNA–protein complexes (86) subdiffuse in the *E. coli* cytoplasm. The reason of subdifussive motion is confined diffusion in a polymer chain and activation processes (87).

We assume in our model that TFs are initially uniformly distributed within a cell. Therefore, our calculations concern a case in which initial position of TF is not correlated with the region of its synthesis (88–90). The assumption is justified by the observation that for fast-growth conditions, considered here, DNA is less densely packed and distribution of LacI does not depend on the spatial location of its encoding gene (89). Also repression strength as a function of intergenic distance is well reproduced by model assuming spatial homogeneity of the repressor (89). Intersegmental jumps that speed up the target location in coiled DNA (91–93) are implicitly embedded in classical model of facilitated diffusion. Since the values of the microscopic and macroscopic dissociation constants are comparable, most of the unbinding events lead to the association to uncorrelated DNA sites.

Our findings also shed light on trade-off between facilitated target location by TFs due to DNA looping, increase of TF concentration and costs of TF production. The increase of TF concentration weaken also the fluctuations in the gene products (94–97). The precise regulation of transcription initiation is easier for dimers with regulation at the dissociation step and direct interactions between dimers. Here, more parameters can be adjusted to reach a gene transfer function that effectively couples the expression of genes to environmental signals. Searching by dimers (98) with further oligomerization at the operator is also preferred in AAA family of TFs (ATPase associated with diverse cellular activities) (Supplementary Table S3). These TFs regulate gene expression in 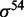-RNAP–dependent promoters. AAA TFs when bound to upstream activating sequences (UAS) function by coupling the energy yielded from ATP hydrolysis to the isomerization of RNAP from closed to open complex. TF oligomerization is required for the formation of functional ATPase and it is promoted by the binding to UAS (99). Therefore, searching by high-oligomeric TF is disadvantageous owing to consumption of ATP when not bound with UAS.

System biology studies of genetic networks require enormous amount of data. Most of them are still unavailable from experiments. Database built here on TF mobilities and searching kinetics can now be used in studies of functioning of these networks with proper temporal resolution to give accurate predictions of gene products fluctuations.

## SUPPLEMENTARY DATA

Supplementary Data are available at NAR Online.

## FUNDING

Ministry of Science of Poland for the Iuventus-Plus program [IP2010 052570 (2011) to M.T.]; National Science Center [DEC1-2011/01/N/ST3/00865 to T.K.]; Foundation for Polish Science for START scholarship [to T.K.]; National Science Center for MAESTRO grant [DEC-2011/02/A/ST3/00143 to R.H.]. Funding for open access charge: MAESTRO grant from National Science Center of Poland [DEC-2011/02/A/ST3/00143].

*Conflict of interest statement*. None declared.

## Supplementary Material

Supplementary Data
