# Supplementary material for: Quantitative influence of macromolecular crowding on gene regulation kinetics
Source: Nucleic Acids Res. 2013 Oct 8;42(2):727–38. doi: 10.1093/nar/gkt907 (PMC3902910; doi:10.1093/nar/gkt907)
Supplement: Supplementary Data [file supp_42_2_727__index.html]

Quantitative influence of macromolecular crowding on gene regulation kinetics — Quantitative influence of macromolecular crowding on gene regulation kinetics — Supplementary Data 

# Quantitative influence of macromolecular crowding on gene regulation kinetics

## Supplementary Data

files

**Files in this Data Supplement:**

- Supplementary Data - pdf file
