# Supplementary material for: Quantitative influence of macromolecular crowding on gene regulation kinetics
Source: Nucleic Acids Res. 2013 Oct 8;42(2):727–38. doi: 10.1093/nar/gkt907 (PMC3902910; doi:10.1093/nar/gkt907)
Supplement: Supplementary Data [file supp_gkt907_nar-01666-v-2013-File010.pdf]

Supplementary information: *Quantitative influence of macromolecular crowding on gene regulation kinetics*

Marcin Tabaka, Tomasz Kalwarczyk, Robert Hołyst

1 Macromolecular crowding on DNA

The concentration of NAPs per cell has been measured [1] in Luria-Bertani broth after 2.3h (it corresponds to the doubling time ~ 20 min [2] and $G = 3.8$ genome equivalents per cell [3]). Here we assumed, that DNA-binding proteins cover DNA on the length equal 2 times their hydrodynamic radius. Lrp forms an open ring structure, in which the DNA is wrapped around the octamer in a nucleosome-like structure [4]. Thus, we calculate that length $2\pi R_h$ of DNA is occupied by Lrp oligomers (similar mode is assumed for DPS). Oligomerisation levels of most NAPs is from [5]. The abundance of RNA polymerase holoenzyme and core complexes are taken from work by Bremer *et al.* [6] for *E. coli* growth rate 2.5 doublings per hour. The number of TFs are calculated from [7] and recalculated for expected number of TFs [8] (we assumed typical values of TF sizes). The number of MukB monomers per cell has been measured to be 400 ± 100 [9] for fast growing cells. Similar value (438 ± 180) has been recently obtained from single-molecule studies [10] but it appeared that only 20% are accumulated in discrete chromosome locations [10, 11]. Mean number of MukB monomers per cell participating in DNA compaction is 82 molecules. The number of cross-links is equal to $M_l = 2\text{MukB} - 1 + \text{SeqA} + \text{MatP}$. The number of cross-links for MukB comes from the following observation. In the presence of ATP, the globular domains of the homodimer associate with each other resulting in tight intramolecular compaction of two DNA chains. MukB dimers can oligomerise forming rosette-like or globular structures [12]. In this way the number of cross-links formed by MukB dimers is 2MukB-1. Recently, it has been recognized that non-classical NAPS such as SeqA, MatP, and SlmA proteins play role in the organizing the chromosome [13]. The concentrations of all ncNAPs are unknown. The number of cross-links formed by the ncNAPs are anticipated from genome conformation capture experiment [14] in the exponentially growing cells. The high clustering between binding sites was attributed to single interaction between two MatP sites and 51 SeqA interaction between sites. Several SeqA loci were involved in interactions with more than single SeqA loci and it is impossible to determine from experiment wheather these multiple interactions appears at the same time. The number of interactions between two loci is 34. No clustering was observed for SlmA binding sites. For the proteins that forms cross-links we assume that they cover approximately 20 bp. MukB, SeqA, and MatP are elongated proteins of length below Kuhn length of DNA [15, 16, 17].

The volume of the cell is taken from [18] ($V_c = 4$ μm^3). The average number of LacI operators is $< O >= 2^{(C(1-x)+T)/\tau}$ [3], where C is the time required to complete replication (42 min), T is time between completion of replication and division (23 min), τ is the doubling time (24 min), and x is the fractional position of LacI operator between the OriC site and terminus (x=0.47). Thus we get, $< O >$=3.7.

2 Calculation of LacI dimer sliding length

We use Monte Carlo simulations to determine the sliding length in the presence of NAPs from the study by Hammar *et al.* [19] on the *in vivo* kinetics of association of *lac* repressor. Hammar *et al.* measured rate constants of LacI binding to two identical operator sites positioned at center-to-center distances 25, 45, 65, 115, 203 bp. They demonstrated correlations for binding of LacI to two operators located at distances smaller than 65 bp. The fit of the experimental data to the model in the absence of NAPs gave value of the sliding length $s_L = \sqrt{2D_1/\Lambda} = 36 \pm 6$ bp. Here we determine the sliding length in the presence of NAPs.

The scheme of the algorithm is shown in Fig. S2. Following work by Berg *et al.* [20] we assume that TF after macroscopic dissociation can rebind to any location on the DNA with the same probability. The availability of the new nonspecific binding site is determined by the arrangement of NAPs in its vicinity. When the site is

obstructed by NAPs the TF can not bind and dissociates macroscopically. In the other case it binds nonspecifically and searches for operator by the sliding. Nonspecifically bound TF to DNA performs one of two exclusive steps: diffusive transition in either direction to the nearest base pair (sliding) or dissociation from DNA surface. The probability of the former is given by $P = 2D_1/(\Lambda + 2D) = 2s^2/(1 + 2s^2)$ and the latter $P_d = 1/(1 + s^2)$, where $s^2 = D_1/\Lambda$. Reflective boundary conditions are considered when TF encounters NAP during sliding.

The sliding length is calculated from the ratio of the mean number T_0 of macroscopic dissociations required for localisation of the single operator to the mean number of T dissociations needed to find one of two operators because it is equal to the ratio of experimentally measured association rates $k/k_0 = T_0/T$. The results of the simulations give the best fit to experimental data for $s^2 = 1000$. All simulations were performed using following parameter values: length of DNA $N = 20$ kbp with operator/operators placed at positions O_1 and O_2, number of NAPs $M = 158$ (vacancy $f = 0.85$). 10^5 simulation realizations were performed to calculate mean number of macroscopic tries that lead to specific binding.

The full event-driven algorithm consists of the following steps:

1. *Initialise*: set s^2, N, $T = 0$, M, O_1, O_2
2. *Arrange NAPs*: for $i = 1$ to M:
   (a) generate a random number $\rho \in (1, N)$:
   (b) if any site from $[\rho, \rho + d]$ has been already occupied, go to step 2. (a)
   (c) if all sites from $[\rho, \rho + d]$ are free, save new position of NAP
3. *Association of TF:* generate a random number $\rho \in (1, N)$
   (a) if any site from $[\rho, \rho + r]$ is occupied by NAP, $T = T + 1$, go to step 2
   (b) if all sites from $[\rho, \rho + r]$ are free, save current position of TF, POS=ρ
4. *Sliding:* if POS is equal to O_1 or O_2, write T, exit; else draw a random number x from the uniform distribution in the unit interval, according to x choose one of three events:
   (a) if POS+1 is free from NAP change the position to POS+1, else to POS-1; (with probability $P_+ = s^2/(1 + 2s^2)$), go to step 4
   (b) if POS-1 is free from NAP change the position to POS-1, else to POS+1; (with probability $P_- = s^2/(1 + 2s^2)$), go to step 4
   (c) *dissociation* (with probability $P_d = 1/(1 + 2s^2)$), $T = T + 1$, go to step 2

Table S1: *E. coli* nucleoid-associated proteins present in the early exponential phase of growth. NAP function: b - bending, l - linking of DNA chains, o - other. ncNAPs - non-classical NAPs.

Protein	oligomerisation	function	level (copies/genome)	Mw [kDa]	r_p [nm]	DNA covered (bp$\times 10^5$)
Rob	monomer	b	2650	33.145	3.04	0.474
Fis	dimer	b	7910	22.480	2.61	1.214
HU	dimer	b	7910	18.761	2.43	1.131
IHF	dimer	b	7250	22.005	2.59	1.104
Lrp	octamer[4]	b	82	151.096	5.52	0.084
H-NS	dimer	o	2650	30.694	2.95	0.460
StpA	dimer	o	3270	30.694	2.95	0.567
Dps	dodecamer[21]	b	133	224.340	6.43	0.158
DnaA	monomer	o	474	52.551	3.64	0.101
IciA	dimer	o	104	66.944	4.01	0.024
MukB	dimer [22]	l	11	-	-	0.005
ncNAPs		l	35-52			0.014-0.021
RNAP	holo		290	459	8.53	0.146
	core		2440	389	7.91	1.135
TF			1400	73	4.49	0.370

Table S2: Specification of *E. coli* transcription factors. List of TF is from *E. coli* EcoCyc [23] and RegulonDB [24] databases. Subscripts denote oligomerisation level of TFs. TFs are grouped according to families they belong to. Molecular weight (Mw, EcoCyc database) of TF is for its oligomeric form. D_3, D_1, and TF search time for single specific-site are calculated in this work. References correspond to works in which oligomerisation level of TF was determined or anticipated. TF with no information about oligomerisation level is assumed to oligomerise in a way that is the most common in its family.

TF	Mw (kDa)	TF family	D_3 (μm^2/s)	D_1 (μm^2/s)	Search time V_n/k (s)	Reference
MarA	15.184	AraC/XylS[1]	16.4	0.33	167	[25]
SoxS	12.911	AraC/XylS	18.7	0.40	157	[26]
Rob	33.145	AraC/XylS	8.2	0.11	240	[27]
$AraC_2$	66.768	AraC/XylS	4.2	0.041	366	[28]
$RhaS_2$	64.63	AraC/XylS	4.3	0.043	358	[29]
$RhaR_2$	71.334	AraC/XylS	3.9	0.037	383	[30]
$CaiF_2$	30.872	AraC/XylS	8.8	0.12	231	[31]
$MelR_2$	69.856	AraC/XylS	4.0	0.038	378	[32]
$XylR_2$	89.738	AraC/XylS	3.1	0.026	453	[33]
$ChbR_2$	65.936	AraC/XylS	4.2	0.042	363	[34]
$FeaR_2$	69.24	AraC/XylS	4.0	0.039	376	
$YdeO_2$	57.45	AraC/XylS	4.8	0.051	331	
$YqhC_2$	71.914	AraC/XylS	3.9	0.036	386	
$YgiV_2$	35.654	AraC/XylS	7.7	0.10	249	
$AdiY_2$	58.014	AraC/XylS	4.8	0.050	334	
$GadW_2$	56.056	AraC/XylS	5.0	0.053	326	[35]
$GadX_2$	63.124	AraC/XylS	4.4	0.044	353	[35]
GadW-GadX	59.59	AraC/XylS	4.7	0.048	339	[35]
$AppY_2$	57.526	AraC/XylS	4.8	0.051	332	
$EnvY_2$	58.038	AraC/XylS	4.8	0.050	334	
$ZntR_2$	32.358	MerR[2]	8.4	0.12	237	[36]
$MlrA_2$	55.092	MerR	5.1	0.054	323	[37]
$CueR_2$	30.47	MerR	8.9	0.13	229	[38]
$SoxR_2$	34.3	MerR	7.9	0.11	244	[39]
$YcgE_2$	56.528	MerR	4.9	0.052	328	
$UidR_2$	43.598	TetR[3]	6.3	0.077	279	[40]
$NemR_2$	44.55	TetR	6.2	0.074	283	[41]
$FabR_2$	53.106	TetR	5.2	0.057	315	[42]
$AcrR_2$	49.534	TetR	5.6	0.064	302	[43]
$BetI_2$	43.63	TetR	6.3	0.076	280	[44]
$RutR_2$	47.376	TetR	5.9	0.068	294	[45]
$EnvR_2$	50.396	TetR	5.5	0.062	305	
$PaaX_2$	70.59	GntR[4]	3.9	0.038	381	[46]
$MngR_2$	56.546	GntR	4.9	0.052	328	
$GlcC_2$	57.652	GntR	4.8	0.051	332	
$McbR_2$	50.302	GntR	5.5	0.062	305	[47]
$PdhR_2$	58.85	GntR	4.7	0.049	337	[48]
$NanR_2$	59.048	GntR	4.7	0.049	337	[49]
$CsiR_2$	49.982	GntR	5.6	0.063	303	

TF	Mw (kDa)	TF family	D_3 (μm^2/s)	D_1 (μm^2/s)	Search time V_n/k (s)	Reference
$FadR_2$	53.938	GntR	5.2	0.056	318	[50]
$LldR_2$	58.332	GntR	4.8	0.050	335	[48]
$UxuR_2$	58.616	GntR	4.8	0.050	336	
$ExuR_2$	59.672	GntR	4.7	0.048	340	
FNR_2	55.934	CRP/FNR	5.0	0.053	326	[51]
CRP_2	47.28	CRP/FNR	5.9	0.068	293	[52]
$YeiL_2$	50.588	CRP/FNR	5.5	0.062	306	[53]
$MarR_2$	32.13	MarR	8.4	0.12	236	
$MprA_2$	41.126	MarR	6.7	0.083	270	[54]
$SlyA_2$	32.706	MarR	8.3	0.12	238	[55]
Fur_2	33.590	Fur	8.1	0.11	241	
Zur_2	38.508	Fur	7.1	0.091	260	[56]
$OmpR_2$	54.708	OmpR	5.1	0.055	321	[57, 58]
$KdpE_2$	50.724	OmpR	5.5	0.061	306	[59]
TorR-TorI	33.911	OmpR	8.0	0.11	243	[60]
$TorR_2$	52.466	OmpR	5.3	0.058	313	[59]
$ArcA_2$	54.584	OmpR	5.1	0.055	321	[61]
$BaeR_2$	55.312	OmpR	5.0	0.054	323	
$CpxR_2$	52.624	OmpR	5.3	0.058	313	[62]
$CreB_2$	52.250	OmpR	5.3	0.059	312	
$PhoB_2$	52.866	OmpR	5.3	0.058	314	[63, 64, 65]
$PhoP_2$	51.070	OmpR	5.4	0.061	308	[66]
$RstA_2$	54.096	OmpR	5.1	0.056	319	
$ArgP_2$	66.944	LysR	4.2	0.041	367	
$DsdC_2$	70.664	LysR	3.9	0.037	381	
$MetR_2$	71.258	LysR	3.9	0.037	383	[67]
$ModE_2$	56.562	LysR	4.9	0.052	328	[68]
$IlvY_2$	66.408	LysR	4.2	0.041	365	[25]
Dan_2	70.630	LysR	3.9	0.037	381	
$BirA_2$	70.624	LysR	3.9	0.037	381	[69]
$CynR_2$	65.922	LysR	4.2	0.042	363	[70]
$HcaR_2$	65.676	LysR	4.2	0.042	362	
$AllS_2$	69.024	LysR	4.0	0.039	375	
$NhaR_2$	68.568	LysR	4.1	0.039	373	
Cbl_2	71.712	LysR	3.9	0.037	385	[71, 72]
Nac_2	65.67	LysR	4.2	0.042	362	[73]
$HdfR_2$	63.492	LysR	4.4	0.044	354	
$GadE_2$	41.198	LysR	6.7	0.083	270	
$LrhA_2$	69.188	LysR	4.0	0.039	375	
$TdcA_2$	69.078	LysR	4.0	0.039	375	
$GcvA_2$	68.804	LysR	4.1	0.039	374	[74]
GcvA-GcvR	55.171	LysR	5.0	0.054	323	[75]
$LysR_2$	68.73	LysR	4.1	0.039	374	
$LeuO_2$	71.39	LysR	3.9	0.037	384	
$XapR_2$	67.254	LysR	4.1	0.040	368	

TF	Mw (kDa)	TF family	D_3 (μm^2/s)	D_1 (μm^2/s)	Search time V_n/k (s)	Reference
$CysB_4$	144.6	LysR	1.9	0.012	669	[76, 77]
$OxyR_4$	137.104	LysR	2.0	0.013	639	[78, 79]
$CytR_2$	75.64	LacI/GalR[5]	3.7	0.034	400	[80]
$TreR_2$	69.062	LacI/GalR	4.0	0.039	375	[81]
$PurR_2$	76.35	LacI/GalR	3.6	0.033	402	[82]
$IdnR_2$	75.334	LacI/GalR	3.7	0.034	398	[83]
$GalR_2$	74.188	LacI/GalR	3.8	0.035	394	[84]
$GalS_2$	74.714	LacI/GalR	3.7	0.034	396	[85]
$AscG_2$	73.888	LacI/GalR	3.8	0.035	393	
$MalI_2$	73.25	LacI/GalR	3.8	0.035	391	
$EbgR_2$	72.42	LacI/GalR	3.8	0.036	387	
$RbsR_2$	73.224	LacI/GalR	3.8	0.035	390	
$GntR_2$	72.884	LacI/GalR	3.8	0.036	389	
$FruR_4$	151.996	LacI/GalR	1.8	0.011	700	[86]
$LacI_4$	154.36	LacI/GalR	1.7	0.011	709	[87]
RcsB-GadE	44.27	LuxR/UhpA	6.2	0.075	282	[88]
$RcsB_2$	47.342	LuxR/UhpA	5.9	0.068	294	[88]
BglJ-RcsB	47.156	LuxR/UhpA	5.9	0.068	293	[89]
RcsBRcsA	47.187	LuxR/UhpA	5.9	0.068	293	[88, 90]
$EvgA_2$	45.38	LuxR/UhpA	6.1	0.072	286	
$NarL_2$	47.854	LuxR/UhpA	5.8	0.067	295	[91, 92]
$NarP_2$	47.150	LuxR/UhpA	5.9	0.068	293	[91, 92]
$CsgD_2$	49.87	LuxR/UhpA	5.6	0.063	303	[93]
$SdiA_2$	56.234	LuxR/UhpA	5.0	0.053	327	[94]
$UhpA_2$	41.779	LuxR/UhpA	6.6	0.081	273	[95]
YoeB-$YefM_2$	28.832	YefM	9.3	0.14	223	[96]
$YefM_2$	18.616	YefM	13.7	0.25	182	[96]
RelE-$RelB_2$	29.367	RelB/DinJ	9.2	0.13	225	[97]
$RelB_2$	18.142	RelB/DinJ	14.1	0.26	180	[97]
$HipB_2$-$HipA_2$	118.584	Cro/C1	2.3	0.017	565	[98]
$HipB_2$	20.032	Cro/C1	12.9	0.23	188	[98]
$MqsA_2MqsR_2$	51.87	Cro/C1	5.4	0.059	311	[99]
$MqsA_2$	29.406	Cro/C1	9.2	0.13	225	[99]
$NadR_2$	94.692	Cro/CI	2.9	0.024	472	[100]
$MhpR_2$	62.644	IclR	4.5	0.045	351	[101]
$KdgR_2$	60.058	IclR	4.6	0.048	341	[102]
$IclR_4$	118.956	IclR	2.3	0.017	566	[103]
$AllR_4$	117.08	IclR	2.3	0.017	559	[104]
$YiaJ_2$	62.134	IclR	4.5	0.046	349	
$DpiA_2$	50.906	CheY	5.5	0.061	307	
$QseB_2$	49.356	CheY	5.6	0.064	301	
$CusR_2$	50.788	CheY	5.5	0.061	306	
$BasR_2$	50.062	CheY	5.6	0.063	304	
$NagC_2$	89.082	NagC/XylR	3.1	0.026	450	[105]
$DgsA_2$	89.632	NagC/XylR	3.1	0.026	452	[106]

TF	Mw (kDa)	TF family	D_3 (μm^2/s)	D_1 (μm^2/s)	Search time V_n/k (s)	Reference
$IscR_2$	34.674	Rrf2	7.9	0.11	246	[107]
$NsrR_2$	31.186	Rrf2	8.7	0.12	232	[108]
$TdcR_2$	17.216	FIS	14.7	0.28	176	
$GutM_2$	25.906	GutM	10.3	0.16	211	
$TrpR_2$	24.71	TrpR	10.7	0.17	207	[109]
$MetJ_2$	24.282	MetJ	10.9	0.17	205	[110]
$MntR_2$	35.28	DtxR	7.7	0.10	248	[111]
$DcuR_2$	54.977	CitAB	5.1	0.054	322	[112]
$ArsR_2$	26.506	ArsR/SmtB	10.1	0.15	214	[113]
$CdaR_2$	87.374	CdaR	3.2	0.027	444	
$MtlR_2$	43.98	YggD	6.3	0.076	281	[114]
$CadC_2$	115.626	ToxR	2.4	0.017	553	[115]
$SgrR_2$	127.95	SgrR	2.1	0.015	602	
$BolA_2$	23.988	BolA	11.0	0.18	204	
$AccB_2$	33.374	biotin carboxylase	8.2	0.11	241	[116]
$LexA_2$	44.716	peptidase s24	6.2	0.074	284	[117]
$AlaS_2$	192.064	tRNA synthetase II	1.3	0.0075	871	[118]
$PutA_2$	287.64	PRO dehydrogenase	0.8	0.0037	1328	[119, 120]
$TyrR_2$	115.312	AAA[6]	2.4	0.017	552	[121]
$PrpR_2$	117.298	AAA	2.3	0.017	560	Tab S3
$RtcR_2$	120.598	AAA	2.3	0.016	573	-
$DhaR_2$	141.124	AAA	1.9	0.013	655	-
MalT	103.118	STAND	2.7	0.021	504	-
$HyfR_2$	150.61	AAA	1.8	0.011	694	-
$FhlA_2$	156.936	AAA	1.7	0.011	720	-
$ZraR_2$	96.788	AAA	2.9	0.023	480	-
$GlrR_2$	98.32	AAA	2.8	0.022	486	-
$NorR_2$	110.472	AAA	2.5	0.019	533	-
$PspF_2$	73.972	AAA	3.8	0.035	393	-
$NtrC_2$	104.51	AAA	2.6	0.020	510	-
$AtoC_2$	104.352	AAA	2.6	0.020	509	-
$AgaR_4$	118.288	DeoR [7]	2.3	0.017	563	[122]
$UlaR_4$	104.408	DeoR	2.6	0.020	509	[123]
$GatR_4$	113.008	DeoR	2.4	0.018	543	
$FucR_4$	109.448	DeoR	2.5	0.019	529	
$GutR_4$	112.944	DeoR	2.4	0.018	542	
$GlpR_4$	112.192	DeoR	2.4	0.018	539	[124]
$DeoR_8$	228.384	DeoR	1.1	0.0056	1037	[125]
$MurR_4$	124.77	AlsR/YebK/YfhH [8]	2.2	0.015	589	[126]
$AlsR_4$	129.448	AlsR/YebK/YfhH	2.1	0.014	608	
$RcnR_4$	40.536	RcnR	6.8	0.085	268	[127]
$AidB_4$	242.36	acyl-CoA dehydrogenase	1.0	0.0050	1103	[128]
$NikR_4$	60.376	CopG/NikR	4.6	0.047	342	[129, 130]
$LsrR_4$	135.188	SorC	2.0	0.013	631	[131]
$FlhD_4$-$FlhC_2$	96.396	FlhD/FlhC	2.9	0.023	478	[132]

TF	Mw (kDa)	TF family	D_3 (μm^2/s)	D_1 (μm^2/s)	Search time V_n/k (s)	Reference
$ArgR_6$	101.96	ArgR	2.7	0.021	500	[133]
$PepA_6$	329.28	peptidase M17	0.7	0.0029	1549	[134]
$YqjI_6$	140.406	PadR	1.9	0.013	652	[135]
$NrdR_8$	137.832	NrdR	2.0	0.013	642	[136]
$AsnC_8$	135.104	AsnC	2.0	0.013	631	[137]
Lrp_8	151.096	AsnC	1.8	0.011	696	[4]

[1] *AraC*-type TFs contain bipartite HTH domains per subunit [138].
[2] All TFs from MerR family are dimers [38].
[3] All TFs from TetR family are dimers [139].
[4] All TFs from GntR family are dimers [140].
[5] Leucine minizipper in the C-terminal region is required for tetramerization of TF from LacI/GalR family [141] (found only for FruR, LacI).
[6] TFs from AAA family search as dimers [142] and oligomerize at an operator.
[7] TFs from DeoR family are higher oligomers - tetramers, octamers with ability of DNA looping[143, 123].
[8] Poorly characterized family of transcription factors, tetramer [126] MurR.

Table S3: AAA family of TFs. HyfR is assumed to be tetramer at operator due to high similarity to FhlA (46%)

TF	Operon	Number of UAS	Oligomerization level at operator	Reference
PrpR	prpR-prpB	2	hexamer	[144]
MalT	malZ	3	tetramer	[145]
	malP	4	tetramer	
	malS	3	tetramer	
	malE	2	tetramer	
	malK	3	tetramer	
HyfR	hyfA	1	tetramer	
FhlA	hycA	2	tetramer	[146]
	hypA	2	tetramer	
	fdhF	2	tetramer	
ZraR	zraS-zraP	2	hexamer	[147]
GlrR	glmY	3	hexamer	
NorR	norR-norV	3	hexamer	[148]
PspF	pspF-pspA	2	hexamer	[149]
NtrC	glnK,nac,glnL	1	hexamer	[142]
	ygjG	2	hexamer	
	astc	3	hexamer	
	glnH	4	hexamer	
	glnA	5	hexamer	
AtoC	atoD	2	hexamer	

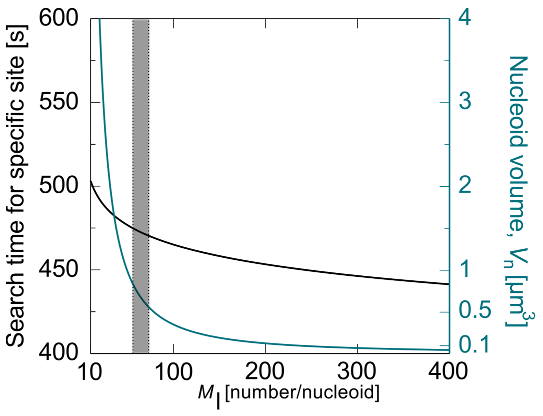

Figure S1: Changes of the LacI dimer searching time for specific site (left axis) and nucleoid volume (right axis) as a function of number of cross-links per nucleoid. The range marked in grey corresponds to the number of cross-links present in early exponential phase of growth [9].

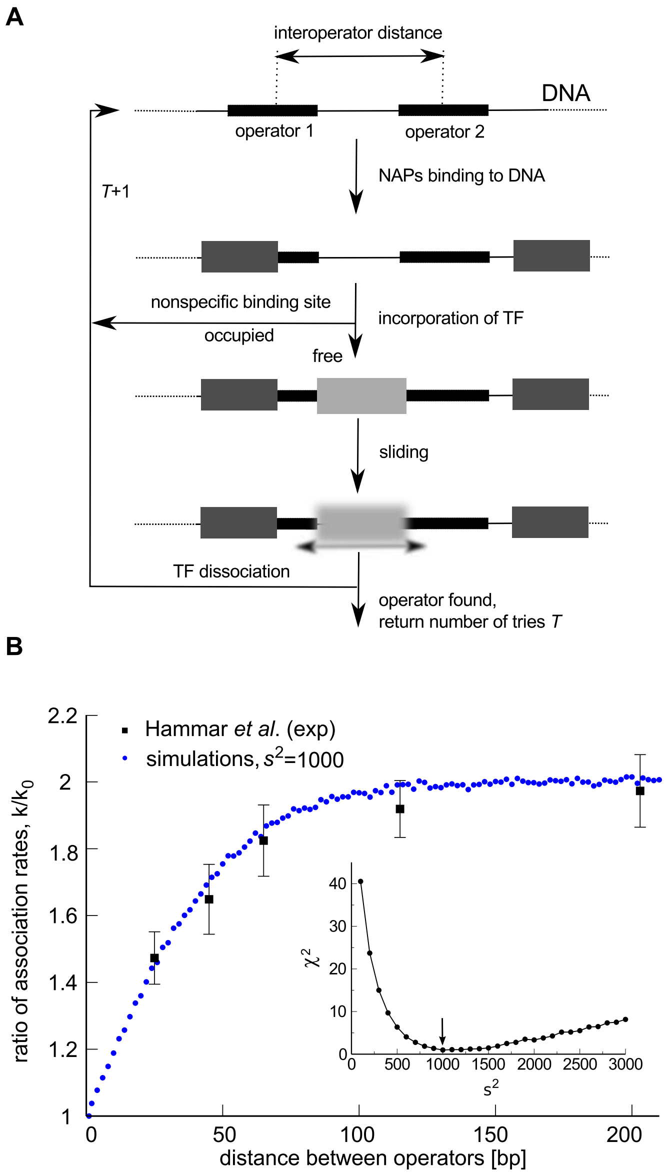

Figure S2: (A) Outline of the simulation algorithm with TF association and sliding in the presence of NAPs. Searching of the operator by TF requires many recurrent macroscopic associations T. Every such event lead to highly correlated scanning of nearby DNA sites by TF sliding. During sliding, TF samples contiguous DNA sites by performing one-dimensional random walk on DNA. (B) Interpretation of the results presented by Hammar *et al.* [19] for experiments with two identical operators (Fig. 2 in Ref. [19]) using model including random arrangement of NAPs on DNA. Ratio of the association rate to two operators to association rate with single operator on DNA as a function of the interoperator distance. Blue dots correspond to the best fit (inset shows χ^2 as a function of s^2 with minimum marked by an arrow) with squared capture distance $s^2 = D_1/\Lambda = 1000$ bp^2 from simulations.

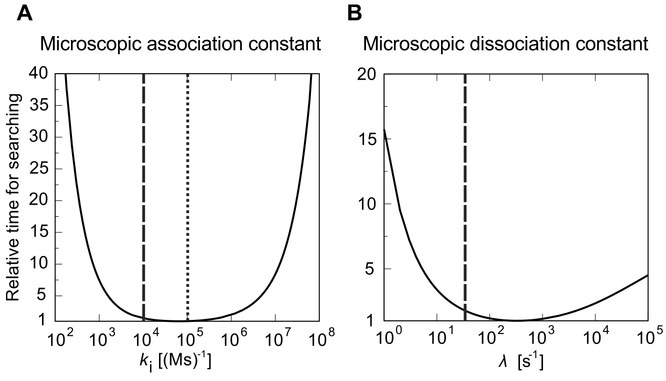

Figure S3: Relative changes of promoter searching by RNAP-σ^{70} complex. (A) Microscopic association constant is predicted to be optimised for fast target location. The times are given with respect to the minimal value. The dashed line corresponds to microscopic constant determined from equilibrium constant $K_{ns}^{\mathrm{RNAP}} = 300$ [150], while dotted one from the value $3000\ \mathrm{M}^{-1}$ [151]. (B) The minimum of searching time as a function of microscopic dissociation rate constant is shifted by one order of magnitude from determined value. The dashed line points to the determined value of microscopic dissociation rate constant.

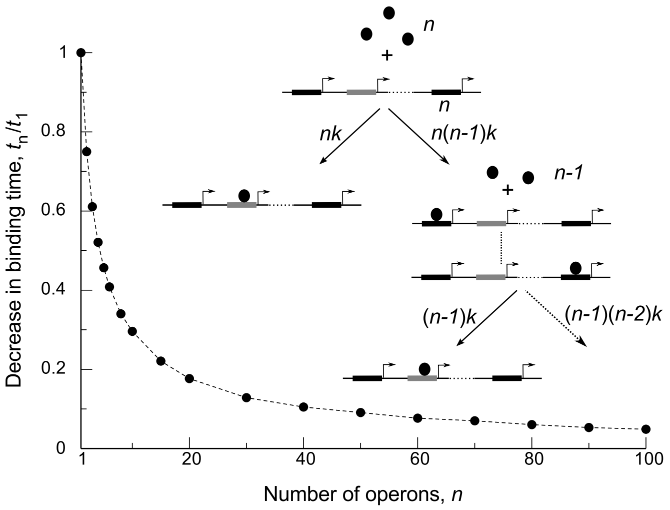

Figure S4: An average binding time to the operator of specific operon decreases with the regulon size. We consider the case in which the total number of TFs is equal to the number of regulated operons (n). Every operon contains single operator. The scheme shows all elementary events that finally lead to binding to the particular operator (grey box). At the first step two outcomes may occur: (1) binding to the specific operator with rate nk because there are n TFs and one specific operator or (2) binding to the rest $(n-1)$ operators with rate $n(n-1)k$. At the following steps the procedure is repeated until specific operator is bound with TF. We assume that unbinding of TF from operator is much slower than TF binding time. The binding time for the case in which $n = 1$ is $t_1 = 1/k$. Values of ratio t_n/t_1 are plotted as a function of the regulon size (number of regulated operons).

References

[1] Ali Azam, T., Iwata, A., Nishimura, A., Ueda, S., and Ishihama, A. (1999) Growth phase-dependent variation in protein composition of the *Escherichia coli* nucleoid. *J. Bacteriol.,* **181**, 6361–6370.

[2] Sezonov, G., Joseleau-Petit, D., and D'Ari, R. (2007) *Escherichia coli* physiology in Luria-Bertani broth. *J. Bacteriol.,* **189**, 8746–8749.

[3] Bremer, H. and Dennis, P. P. Modulation of chemical composition and other parameters of the cell by growth rate. Vol. 2 of Escherichia coli and Salmonella thyphymurium: Cellular and molecular biology, pp. 1553–1569 ASM Press. Washington DC (1996).

[4] de los Rios, S. and Perona, J. J. (2007) Structure of the *Escherichia coli* leucine-responsive regulatory protein Lrp reveals a novel octameric assembly. *J. Mol. Biol.,* **366**, 1589–1602.

[5] Azam, T. A. and Ishihama, A. (1999) Twelve species of the nucleoid-associated protein from *Escherichia coli*. Sequence recognition specificity and DNA binding affinity. *J. Biol. Chem.,* **274**, 33105–33113.

[6] Bremer, H., Dennis, P., and Ehrenberg, M. (2003) Free RNA polymerase and modeling global transcription in *Escherichia coli*. *Biochimie,* **85**, 597–609.

[7] Taniguchi, Y., Choi, P. J., Li, G. W., Chen, H., Babu, M., Hearn, J., Emili, A., and Xie, X. S. (2010) Quantifying *E. coli* proteome and transcriptome with single-molecule sensitivity in single cells. *Science,* **329**, 533–538.

[8] Madan Babu, M. and Teichmann, S. A. (2003) Evolution of transcription factors and the gene regulatory network in *Escherichia coli*. *Nucleic Acids Res.,* **31**, 1234–1244.

[9] Petrushenko, Z. M., Lai, C. H., and Rybenkov, V. V. (2006) Antagonistic interactions of kleisins and DNA with bacterial Condensin MukB. *J. Biol. Chem.,* **281**, 34208–34217.

[10] Badrinarayanan, A., Reyes-Lamothe, R., Uphoff, S., Leake, M. C., and Sherratt, D. J. (2012) *In vivo* architecture and action of bacterial structural maintenance of chromosome proteins. *Science,* **338**, 528–531.

[11] Kleine Borgmann, L. A., Ries, J., Ewers, H., Ulbrich, M. H., and Graumann, P. L. (2013) The bacterial SMC complex displays two distinct modes of interaction with the chromosome. *Cell Rep.,* **3**, 1483–1492.

[12] Chen, N., Zinchenko, A. A., Yoshikawa, Y., Araki, S., Adachi, S., Yamazoe, M., Hiraga, S., and Yoshikawa, K. (2008) ATP-induced shrinkage of DNA with MukB protein and the MukBEF complex of *Escherichia coli*. *J. Bacteriol.,* **190**, 3731–3737.

[13] Dame, R. T., Kalmykowa, O. J., and Grainger, D. C. (2011) Chromosomal macrodomains and associated proteins: implications for DNA organization and replication in gram negative bacteria. *PLoS Genet.,* **7**, e1002123.

[14] Cagliero, C., Grand, R. S., Jones, M. B., Jin, D. J., and O'Sullivan, J. M. (2013) Genome conformation capture reveals that the *Escherichia coli* chromosome is organized by replication and transcription. *Nucleic Acids Res.,* **41**, 6058–6071.

[15] Matoba, K., Yamazoe, M., Mayanagi, K., Morikawa, K., and Hiraga, S. (2005) Comparison of MukB homodimer versus MukBEF complex molecular architectures by electron microscopy reveals a higher-order multimerization. *Biochem. Biophys. Res. Commun.,* **333**, 694–702.

[16] Waldminghaus, T. and Skarstad, K. (2009) The *Escherichia coli* SeqA protein. *Plasmid,* **61**(3), 141–150.

[17] Dupaigne, P., Tonthat, N. K., Espéli, O., Whitfill, T., Boccard, F., and Schumacher, M. A. (2012) Molecular basis for a protein-mediated DNA-bridging mechanism that functions in condensation of the *E. coli* chromosome. *Mol. Cell,* **48**, 560–571.

[18] Volkmer, B. and Heinemann, M. (2011) Condition-dependent cell volume and concentration of *Escherichia coli* to facilitate data conversion for systems biology modeling. *PLoS One,* **6**, e23126.

[19] Hammar, P., Leroy, P., Mahmutovic, A., Marklund, E. G., Berg, O. G., and Elf, J. (2012) The *lac* repressor displays facilitated diffusion in living cells. *Science,* **336**, 1595–1598.

[20] Berg, O. G., Winter, R. B., and von Hippel, P. H. (1981) Diffusion-driven mechanisms of protein translocation on nucleic acids. 1. Models and theory. *Biochemistry,* **20**, 6929–6948.

[21] Grant, R. A., Filman, D. J., Finkel, S. E., Kolter, R., and Hogle, J. M. (1998) The crystal structure of Dps, a ferritin homolog that binds and protects DNA. *Nat. Struct. Biol.,* **5**, 294–303.

[22] Niki, H., Imamura, R., Kitaoka, M., Yamanaka, K., Ogura, T., and Hiraga, S. (1992) *E. coli* MukB protein involved in chromosome partition forms a homodimer with a rod-and-hinge structure having DNA binding and ATP/GTP binding activities. *EMBO J.,* **11**, 5101–5109.

[23] Keseler, I. M., Collado-Vides, J., Santos-Zavaleta, A., Peralta-Gil, M., Gama-Castro, S., Muñiz-Rascado, L., Bonavides-Martinez, C., Paley, S., Krummenacker, M., Altman, T., Kaipa, P., Spaulding, A., Pacheco, J., Latendresse, M., Fulcher, C., Sarker, M., Shearer, A. G., Mackie, A., Paulsen, I., Gunsalus, R. P., and Karp, P. D. (2011) EcoCyc: a comprehensive database of *Escherichia coli* biology. *Nucleic Acids Res.,* **39**, 583–590.

[24] Gama-Castro, S., Salgado, H., Peralta-Gil, M., Santos-Zavaleta, A., Muñiz-Rascado, L., Solano-Lira, H., Jimenez-Jacinto, V., Weiss, V., García-Sotelo, J. S., López-Fuentes, A., Porrón-Sotelo, L., Alquicira-Hernández, S., Medina-Rivera, A., Martínez-Flores, I., Alquicira-Hernández, K., Martínez-Adame, R., Bonavides-Martínez, C., Miranda-Ríos, J., Huerta, A. M., Mendoza-Vargas, A., Collado-Torres, L., Taboada, B., Vega-Alvarado, L., Olvera, M., Olvera, L., Grande, R., Morett, E., and Collado-Vides, J. (2011) RegulonDB version 7.0: transcriptional regulation of *Escherichia coli* K-12 integrated within genetic sensory response units (Gensor Units). *Nucleic Acids Res.,* **39**, 98–105.

[25] Rhee, K. Y., Senear, D. F., and Hatfield, G. W. (1998) Activation of gene expression by a ligand-induced conformational change of a protein-DNA complex. *J. Biol. Chem.,* **273**, 11257–11266.

[26] Jair, K. W., Fawcett, W. P., Fujita, N., Ishihama, A., and Wolf, R. E. (1996) Ambidextrous transcriptional activation by SoxS: requirement for the C-terminal domain of the RNA polymerase alpha subunit in a subset of *Escherichia coli* superoxide-inducible genes. *Mol. Microbiol.,* **19**, 307–317.

[27] Kwon, H. J., Bennik, M. H., Demple, B., and Ellenberger, T. (2000) Crystal structure of the *Escherichia coli* Rob transcription factor in complex with DNA. *Nat. Struct. Biol.,* **7**, 424–430.

[28] Soisson, S. M., MacDougall-Shackleton, B., Schleif, R., and Wolberger, C. (1997) Structural basis for ligand-regulated oligomerization of AraC. *Science,* **276**, 421–425.

[29] Bhende, P. M. and Egan, S. M. (1999) Amino acid-DNA contacts by RhaS: an AraC family transcription activator. *J. Bacteriol.,* **181**, 5185–5192.

[30] Wickstrum, J. R. and Egan, S. M. (2004) Amino acid contacts between sigma 70 domain 4 and the transcription activators RhaS and RhaR. *J. Bacteriol.,* **186**, 6277–6285.

[31] Buchet, A., Nasser, W., Eichler, K., and Mandrand-Berthelot, M. A. (1999) Positive co-regulation of the *Escherichia coli* carnitine pathway cai and fix operons by CRP and the CaiF activator. *Mol. Microbiol.,* **34**, 562–575.

[32] Grainger, D. C., Belyaeva, T. A., Lee, D. J., Hyde, E. I., and Busby, S. J. (2003) Binding of the *Escherichia coli* MelR protein to the melAB promoter: orientation of MelR subunits and investigation of MelR-DNA contacts. *Mol. Microbiol.,* **48**, 335–348.

[33] Song, S. and Park, C. (1997) Organization and regulation of the D-xylose operons in *Escherichia coli* K-12: XylR acts as a transcriptional activator. *J. Bacteriol.,* **179**, 7025–7032.

[34] Plumbridge, J. and Pellegrini, O. (2004) Expression of the chitobiose operon of *Escherichia coli* is regulated by three transcription factors: NagC, ChbR and CAP. *Mol. Microbiol.,* **52**, 437–449.

[35] Ma, Z., Richard, H., Tucker, D. L., Conway, T., and Foster, J. W. (2002) Collaborative regulation of *Escherichia coli* glutamate-dependent acid resistance by two AraC-like regulators, GadX and GadW (YhiW). *J. Bacteriol.,* **184**, 7001–7012.

[36] Changela, A., Chen, K., Xue, Y., Holschen, J., Outten, C. E., O'Halloran, T. V., and Mondragón, A. (2003) Molecular basis of metal-ion selectivity and zeptomolar sensitivity by CueR. *Science,* **301**, 1383–1387.

[37] Ogasawara, H., Yamamoto, K., and Ishihama, A. (2010) Regulatory role of MlrA in transcription activation of csgD, the master regulator of biofilm formation in *Escherichia coli*. *FEMS Microbiol. Lett.,* **312**, 160–168.

[38] Andoy, N. M., Sarkar, S. K., Wang, Q., Panda, D., Benítez, J. J., Kalininskiy, A., and Chen, P. (2009) Single-molecule study of metalloregulator CueR-DNA interactions using engineered Holliday junctions. *Biophys. J.,* **97**, 844–852.

[39] Hidalgo, E., Bollinger, J. M., Bradley, T. M., Walsh, C. T., and Demple, B. (1995) Binuclear [2Fe-2S] clusters in the *Escherichia coli* SoxR protein and role of the metal centers in transcription. *J. Biol. Chem.,* **270**, 20908–20914.

[40] Blanco, C., Ritzenthaler, P., and Mata-Gilsinger, M. (1986) Negative dominant mutations of the uidR gene in *Escherichia coli*: genetic proof for a cooperative regulation of uidA expression. *Genetics,* **112**, 173–182.

[41] Umezawa, Y., Shimada, T., Kori, A., Yamada, K., and Ishihama, A. (2008) The uncharacterized transcription factor YdhM is the regulator of the nemA gene, encoding N-ethylmaleimide reductase. *J. Bacteriol.,* **190**, 5890–5897.

[42] Zhu, K., Zhang, Y. M., and Rock, C. O. (2009) Transcriptional regulation of membrane lipid homeostasis in *Escherichia coli*. *J. Biol. Chem.,* **284**, 34880–34888.

[43] Li, M., Gu, R., Su, C. C., Routh, M. D., Harris, K. C., Jewell, E. S., McDermott, G., and Yu, E. W. (2007) Crystal structure of the transcriptional regulator AcrR from *Escherichia coli*. *J. Mol. Biol.,* **374**, 591–603.

[44] Rokenes, T. P., Lamark, T., and Strom, A. R. (1996) DNA-binding properties of the BetI repressor protein of *Escherichia coli*: the inducer choline stimulates BetI-DNA complex formation. *J. Bacteriol.,* **178**, 1663–1670.

[45] Shimada, T., Hirao, K., Kori, A., Yamamoto, K., and Ishihama, A. (2007) RutR is the uracil/thymine-sensing master regulator of a set of genes for synthesis and degradation of pyrimidines. *Mol. Microbiol.,* **66**, 744–757.

[46] Kim, H. S., Kang, T. S., Hyun, J. S., and Kang, H. S. (2004) Regulation of penicillin G acylase gene expression in *Escherichia coli* by repressor PaaX and the cAMP-cAMP receptor protein complex. *J. Biol. Chem.,* **279**, 33253–33262.

[47] Beraud, M., Kolb, A., Monteil, V., D'Alayer, J., and Norel, F. (2010) A proteomic analysis reveals differential regulation of the σ(S)-dependent yciGFE(katN) locus by YncC and H-NS in *Salmonella* and *Escherichia coli* K-12. *Mol. Cell Proteomics,* **9**, 2601–2616.

[48] Aguilera, L., Campos, E., Giménez, R., Badía, J., Aguilar, J., and Baldoma, L. (2008) Dual role of LldR in regulation of the lldPRD operon, involved in L-lactate metabolism in *Escherichia coli*. *J. Bacteriol.,* **190**, 2997–3005.

[49] Kalivoda, K. A., Steenbergen, S. M., Vimr, E. R., and Plumbridge, J. (2003) Regulation of sialic acid catabolism by the DNA binding protein NanR in *Escherichia coli*. *J. Bacteriol.,* **185**, 4806–4815.

[50] Raman, N., Black, P. N., and DiRusso, C. C. (1997) Characterization of the fatty acid-responsive transcription factor FadR. Biochemical and genetic analyses of the native conformation and functional domains. *J. Biol. Chem.,* **272**, 30645–30650.

[51] Moore, L. J. and Kiley, P. J. (2001) Characterization of the dimerization domain in the FNR transcription factor. *J. Biol. Chem.,* **276**, 45744–45750.

[52] McKay, D. B., Weber, I. T., and Steitz, T. A. (1982) Structure of catabolite gene activator protein at 2.9-A resolution. Incorporation of amino acid sequence and interactions with cyclic AMP. *J. Biol. Chem.,* **257**, 9518–9524.

[53] Anjum, M. F., Green, J., and Guest, J. R. (2000) YeiL, the third member of the CRP-FNR family in *Escherichia coli*. *Microbiology,* **146 Pt 12**, 3157–3170.

[54] Brooun, A., Tomashek, J. J., and Lewis, K. (1999) Purification and ligand binding of EmrR, a regulator of a multidrug transporter. *J. Bacteriol.,* **181**, 5131–5133.

[55] Wu, R. Y., Zhang, R. G., Zagnitko, O., Dementieva, I., Maltzev, N., Watson, J. D., Laskowski, R., Gornicki, P., and Joachimiak, A. (2003) Crystal structure of *Enterococcus faecalis* SlyA-like transcriptional factor. *J. Biol. Chem.,* **278**, 20240–20244.

[56] Patzer, S. I. and Hantke, K. (2000) The zinc-responsive regulator Zur and its control of the znu gene cluster encoding the ZnuABC zinc uptake system in *Escherichia coli*. *J. Biol. Chem.,* **275**, 24321–24332.

[57] Harrison-McMonagle, P., Denissova, N., Ebright, R., Stock, A., et al. (1999) Orientation of OmpR monomers within an OmpR: DNA complex determined by DNA affinity cleaving. *J. Mol. Biol.,* **285**, 555–566.

[58] Bachhawat, P. and Stock, A. M. (2007) Crystal structures of the receiver domain of the response regulator PhoP from *Escherichia coli* in the absence and presence of the phosphoryl analog beryllofluoride. *J. Bacteriol.,* **189**, 5987–5995.

[59] Toro-Roman, A., Wu, T., and Stock, A. M. (2005) A common dimerization interface in bacterial response regulators KdpE and TorR. *Protein Sci.,* **14**, 3077–3088.

[60] Ansaldi, M., Théraulaz, L., and Méjean, V. (2004) TorI, a response regulator inhibitor of phage origin in *Escherichia coli*. *Proc. Natl. Acad. Sci. USA,* **101**, 9423–9428.

[61] Toro-Roman, A., Mack, T., and Stock, A. (2005) Structural Analysis and Solution Studies of the Activated Regulatory Domain of the Response Regulator ArcA: A Symmetric Dimer Mediated by the [alpha] 4-[beta] 5-[alpha] 5 Face. *J. Mol. Biol.,* **349**, 11–26.

[62] Jubelin, G., Vianney, A., Beloin, C., Ghigo, J. M., Lazzaroni, J. C., Lejeune, P., and Dorel, C. (2005) CpxR/OmpR interplay regulates curli gene expression in response to osmolarity in *Escherichia coli*. *J. Bacteriol.,* **187**, 2038–2049.

[63] Fiedler, U. and Weiss, V. (1995) A common switch in activation of the response regulators NtrC and PhoB: phosphorylation induces dimerization of the receiver modules. *EMBO J.,* **14**, 3696–3705.

[64] Blanco, A. G., Sola, M., Gomis-Rüth, F. X., and Coll, M. (2002) Tandem DNA recognition by PhoB, a two-component signal transduction transcriptional activator. *Structure,* **10**, 701–713.

[65] Mack, T. R., Gao, R., and Stock, A. M. (2009) Probing the roles of the two different dimers mediated by the receiver domain of the response regulator PhoB. *J. Mol. Biol.,* **389**, 349–364.

[66] Yamamoto, K., Ogasawara, H., Fujita, N., Utsumi, R., and Ishihama, A. (2002) Novel mode of transcription regulation of divergently overlapping promoters by PhoP, the regulator of two-component system sensing external magnesium availability. *Mol. Microbiol.,* **45**, 423–438.

[67] Maxon, M. E., Wigboldus, J., Brot, N., and Weissbach, H. (1990) Structure-function studies on *Escherichia coli* MetR protein, a putative prokaryotic leucine zipper protein. *Proc. Natl. Acad. Sci. USA,* **87**, 7076–7079.

[68] Anderson, L. A., Palmer, T., Price, N. C., Bornemann, S., Boxer, D. H., and Pau, R. N. (1997) Characterisation of the molybdenum-responsive ModE regulatory protein and its binding to the promoter region of the modABCD (molybdenum transport) operon of *Escherichia coli*. *Eur. J. Biochem.,* **246**, 119–126.

[69] Weaver, L. H., Kwon, K., Beckett, D., and Matthews, B. W. (2001) Corepressor-induced organization and assembly of the biotin repressor: a model for allosteric activation of a transcriptional regulator. *Proc. Natl. Acad. Sci. USA,* **98**, 6045–6050.

[70] Lamblin, A. F. and Fuchs, J. A. (1994) Functional analysis of the *Escherichia coli* K-12 *cyn* operon transcriptional regulation. *J. Bacteriol.,* **176**, 6613–6622.

[71] Stec, E., Witkowska, M., Hryniewicz, M. M., Brzozowski, A. M., Wilkinson, A. J., and Bujacz, G. D. (2004) Crystallization and preliminary crystallographic studies of the cofactor-binding domain of the LysR-type transcriptional regulator Cbl from *Escherichia coli*. *Acta Crystallogr. D Biol. Crystallogr.,* **60**, 1654–1657.

[72] Stec, E., Witkowska-Zimny, M., Hryniewicz, M. M., Neumann, P., Wilkinson, A. J., Brzozowski, A. M., Verma, C. S., Zaim, J., Wysocki, S., and Bujacz, G. D. (2006) Structural basis of the sulphate starvation response in *E. coli*: crystal structure and mutational analysis of the cofactor-binding domain of the Cbl transcriptional regulator. *J. Mol. Biol.,* **364**, 309–322.

[73] Rosario, C. J., Frisch, R. L., and Bender, R. A. (2010) The LysR-type nitrogen assimilation control protein forms complexes with both long and short DNA binding sites in the absence of coeffectors. *J. Bacteriol.,* **192**, 4827–4833.

[74] Wilson, R. L., Urbanowski, M. L., and Stauffer, G. V. (1995) DNA binding sites of the LysR-type regulator GcvA in the gcv and gcvA control regions of *Escherichia coli*. *J. Bacteriol.,* **177**, 4940–4946.

[75] Ghrist, A. C., Heil, G., and Stauffer, G. V. (2001) GcvR interacts with GcvA to inhibit activation of the *Escherichia coli* glycine cleavage operon. *Microbiology,* **147**, 2215–2221.

[76] Hryniewicz, M. M. and Kredich, N. M. (1994) Stoichiometry of binding of CysB to the cysJIH, cysK, and cysP promoter regions of *Salmonella typhimurium*. *J. Bacteriol.,* **176**, 3673–3682.

[77] Lochowska, A., Iwanicka-Nowicka, R., Plochocka, D., and Hryniewicz, M. M. (2001) Functional dissection of the LysR-type CysB transcriptional regulator. Regions important for DNA binding, inducer response, oligomerization, and positive control. *J. Biol. Chem.,* **276**, 2098–2107.

[78] Kullik, I., Stevens, J., Toledano, M. B., and Storz, G. (1995) Mutational analysis of the redox-sensitive transcriptional regulator OxyR: regions important for DNA binding and multimerization. *J. Bacteriol.,* **177**, 1285–1291.

[79] Toledano, M. B., Kullik, I., Trinh, F., Baird, P. T., Schneider, T. D., and Storz, G. (1994) Redox-dependent shift of OxyR-DNA contacts along an extended DNA-binding site: a mechanism for differential promoter selection. *Cell,* **78**, 897–909.

[80] Moody, C. L., Tretyachenko-Ladokhina, V., Laue, T. M., Senear, D. F., and Cocco, M. J. (2011) Multiple conformations of the cytidine repressor DNA-binding domain coalesce to one upon recognition of a specific DNA surface. *Biochemistry,* **50**, 6622–6632.

[81] Hars, U., Horlacher, R., Boos, W., Welte, W., and Diederichs, K. (1998) Crystal structure of the effector-binding domain of the trehalose-repressor of *Escherichia coli*, a member of the LacI family, in its complexes with inducer trehalose-6-phosphate and noninducer trehalose. *Protein Sci.,* **7**, 2511–2521.

[82] Choi, K. Y. and Zalkin, H. (1992) Structural characterization and corepressor binding of the *Escherichia coli* purine repressor. *J. Bacteriol.,* **174**, 6207–6214.

[83] Bausch, C. L. The L-idonic acid pathway of *Escherichia Coli* PhD thesis School of The Ohio State University (2003).

[84] Irani, M. H., Orosz, L., and Adhya, S. (1983) A control element within a structural gene: the *gal* operon of *Escherichia coli*. *Cell,* **32**, 783–788.

[85] Geanacopoulos, M. and Adhya, S. (1997) Functional characterization of roles of GalR and GalS as regulators of the gal regulon. *J. Bacteriol.,* **179**, 228–234.

[86] Cortay, J. C., Nègre, D., Scarabel, M., Ramseier, T. M., Vartak, N. B., Reizer, J., Saier, M. H., and Cozzone, A. J. (1994) *In vitro* asymmetric binding of the pleiotropic regulatory protein, FruR, to the *ace* operator controlling glyoxylate shunt enzyme synthesis. *J. Biol. Chem.,* **269**, 14885–14891.

[87] Friedman, A. M., Fischmann, T. O., and Steitz, T. A. (1995) Crystal structure of *lac* repressor core tetramer and its implications for DNA looping. *Science,* **268**, 1721–1727.

[88] Carballès, F., Bertrand, C., Bouché, J. P., and Cam, K. (1999) Regulation of *Escherichia coli* cell division genes ftsA and ftsZ by the two-component system rcsC-rcsB. *Mol. Microbiol.,* **34**, 442–450.

[89] Madhusudan, S., Paukner, A., Klingen, Y., and Schnetz, K. (2005) Independent regulation of H-NS-mediated silencing of the bgl operon at two levels: upstream by BglJ and LeuO and downstream by DnaKJ. *Microbiology,* **151**, 3349–3359.

[90] Wehland, M. and Bernhard, F. (2000) The RcsAB box. Characterization of a new operator essential for the regulation of exopolysaccharide biosynthesis in enteric bacteria. *J. Biol. Chem.,* **275**, 7013–7020.

[91] Baikalov, I., Schröder, I., Kaczor-Grzeskowiak, M., Cascio, D., Gunsalus, R. P., and Dickerson, R. E. (1998) NarL dimerization? Suggestive evidence from a new crystal form. *Biochemistry,* **37**, 3665–3676.

[92] Maris, A. E., Sawaya, M. R., Kaczor-Grzeskowiak, M., Jarvis, M. R., Bearson, S. M., Kopka, M. L., Schröder, I., Gunsalus, R. P., and Dickerson, R. E. (2002) Dimerization allows DNA target site recognition by the NarL response regulator. *Nature Struct. Biol.,* **9**, 771–778.

[93] Brombacher, E., Dorel, C., Zehnder, A. J., and Landini, P. (2003) The curli biosynthesis regulator CsgD co-ordinates the expression of both positive and negative determinants for biofilm formation in *Escherichia coli*. *Microbiology,* **149**, 2847–2857.

[94] Yao, Y., Martinez-Yamout, M. A., Dickerson, T. J., Brogan, A. P., Wright, P. E., and Dyson, H. J. (2006) Structure of the *Escherichia coli* quorum sensing protein SdiA: activation of the folding switch by acyl homoserine lactones. *J. Mol. Biol.,* **355**, 262–273.

[95] Olekhnovich, I. N. and Kadner, R. J. (2002) Mutational scanning and affinity cleavage analysis of UhpA-binding sites in the *Escherichia coli* uhpT promoter. *J. Bacteriol.,* **184**, 2682–2691.

[96] Kedzierska, B., Lian, L. Y., and Hayes, F. (2007) Toxin-antitoxin regulation: bimodal interaction of YefM-YoeB with paired DNA palindromes exerts transcriptional autorepression. *Nucleic Acids. Res.,* **35**, 325–339.

[97] Li, G. Y., Zhang, Y., Inouye, M., and Ikura, M. (2008) Structural mechanism of transcriptional autorepression of the *Escherichia coli* RelB/RelE antitoxin/toxin module. *J. Mol. Biol.,* **380**, 107–119.

[98] Schumacher, M. A., Piro, K. M., Xu, W., Hansen, S., Lewis, K., and Brennan, R. G. (2009) Molecular mechanisms of HipA-mediated multidrug tolerance and its neutralization by HipB. *Science,* **323**, 396–401.

[99] Brown, B., Grigoriu, S., Kim, Y., Arruda, J., Davenport, A., Wood, T., Peti, W., and Page, R. (2009) Three dimensional structure of the MqsR: MqsA complex: a novel TA pair comprised of a toxin homologous to RelE and an antitoxin with unique properties. *PLoS pathogens,* **5**, e1000706.

[100] Penfound, T. and Foster, J. W. (1999) NAD-dependent DNA-binding activity of the bifunctional NadR regulator of *Salmonella typhimurium*. *J. Bacteriol.,* **181**, 648–655.

[101] Manso, I., Torres, B., Andreu, J. M., Menéndez, M., Rivas, G., Alfonso, C., Díaz, E., García, J. L., and Galán, B. (2009) 3-Hydroxyphenylpropionate and phenylpropionate are synergistic activators of the MhpR transcriptional regulator from *Escherichia coli*. *J. Biol. Chem.,* **284**, 21218–21228.

[102] Nasser, W., Reverchon, S., Condemine, G., and Robert-Baudouy, J. (1994) Specific interactions of *Erwinia chrysanthemi* KdgR repressor with different operators of genes involved in pectinolysis. *J. Mol. Biol.,* **236**, 427–440.

[103] Lorca, G. L., Ezersky, A., Lunin, V. V., Walker, J. R., Altamentova, S., Evdokimova, E., Vedadi, M., Bochkarev, A., and Savchenko, A. (2007) Glyoxylate and pyruvate are antagonistic effectors of the *Escherichia coli* IclR transcriptional regulator. *J. Biol. Chem.,* **282**, 16476–16491.

[104] Hasegawa, A., Ogasawara, H., Kori, A., Teramoto, J., and Ishihama, A. (2008) The transcription regulator AllR senses both allantoin and glyoxylate and controls a set of genes for degradation and reutilization of purines. *Microbiology,* **154**, 3366–3378.

[105] Pennetier, C., Domínguez-Ramírez, L., and Plumbridge, J. (2008) Different regions of Mlc and NagC, homologous transcriptional repressors controlling expression of the glucose and N-acetylglucosamine phosphotransferase systems in *Escherichia coli*, are required for inducer signal recognition. *Mol. Microbiol.,* **67**, 364–377.

[106] Schiefner, A., Gerber, K., Seitz, S., Welte, W., Diederichs, K., and Boos, W. (2005) The crystal structure of Mlc, a global regulator of sugar metabolism in *Escherichia coli*. *J. Biol. Chem.,* **280**, 29073–29079.

[107] Nesbit, A. D., Giel, J. L., Rose, J. C., and Kiley, P. J. (2009) Sequence-specific binding to a subset of IscR-regulated promoters does not require IscR Fe-S cluster ligation. *J. Mol. Biol.,* **387**, 28–41.

[108] Tucker, N. P., Hicks, M. G., Clarke, T. A., Crack, J. C., Chandra, G., Le Brun, N. E., Dixon, R., and Hutchings, M. I. (2008) The transcriptional repressor protein NsrR senses nitric oxide directly via a [2Fe-2S] cluster. *PLoS One,* **3**, e3623.

[109] Joachimiak, A., Kelley, R. L., Gunsalus, R. P., Yanofsky, C., and Sigler, P. B. (1983) Purification and characterization of *trp* aporepressor. *Proc. Natl. Acad. Sci. USA,* **80**, 668–672.

[110] Smith, A. A., Greene, R. C., Kirby, T. W., and Hindenach, B. R. (1985) Isolation and characterization of the product of the methionine-regulatory gene metJ of *Escherichia coli* K-12. *Proc. Natl. Acad. Sci. USA,* **82**, 6104–6108.

[111] Tanaka, T., Shinkai, A., Bessho, Y., Kumarevel, T., and Yokoyama, S. (2009) Crystal structure of the manganese transport regulatory protein from *Escherichia coli*. *Proteins,* **77**, 741–746.

[112] Abo-Amer, A. E., Munn, J., Jackson, K., Aktas, M., Golby, P., Kelly, D. J., and Andrews, S. C. (2004) DNA interaction and phosphotransfer of the C4-dicarboxylate-responsive DcuS-DcuR two-component regulatory system from *Escherichia coli*. *J. Bacteriol.,* **186**, 1879–1889.

[113] Xu, C. and Rosen, B. P. (1997) Dimerization is essential for DNA binding and repression by the ArsR metalloregulatory protein of *Escherichia coli*. *J. Biol. Chem.,* **272**, 15734–15738.

[114] Tan, K., Clancy, S., Borovilos, M., Zhou, M., Hörer, S., Moy, S., Volkart, L. L., Sassoon, J., Baumann, U., and Joachimiak, A. (2009) The mannitol operon repressor MtlR belongs to a new class of transcription regulators in bacteria. *J. Biol. Chem.,* **284**, 36670–36679.

[115] Kuper, C. and Jung, K. (2005) CadC-mediated activation of the cadBA promoter in *Escherichia coli*. *J. Mol. Microbiol. Biotechnol.,* **10**, 26–39.

[116] James, E. S. and Cronan, J. E. (2004) Expression of two *Escherichia coli* acetyl-CoA carboxylase subunits is autoregulated. *J. Biol. Chem.,* **279**, 2520–2527.

[117] Mohana-Borges, R., Pacheco, A. B., Sousa, F. J., Foguel, D., Almeida, D. F., and Silva, J. L. (2000) LexA repressor forms stable dimers in solution. The role of specific DNA in tightening protein-protein interactions. *J. Biol. Chem.,* **275**, 4708–4712.

[118] Sood, S. M., Slattery, C. W., Filley, S. J., Wu, M. X., and Hill, K. A. (1996) Further characterization of *Escherichia coli* alanyl-tRNA synthetase. *Arch. Biochem. Biophys.,* **328**, 295–301.

[119] Brown, E. D. and Wood, J. M. (1992) Redesigned purification yields a fully functional PutA protein dimer from *Escherichia coli*. *J. Biol. Chem.,* **267**, 13086–13092.

[120] Singh, R., Larson, J., Zhu, W., Rambo, R., Hura, G., Becker, D., and Tanner, J. (2011) Small-angle X-ray scattering studies of the oligomeric state and quaternary structure of the trifunctional proline utilization A (PutA) flavoprotein from *Escherichia coli*. *J. Biol. Chem.,* **286**, 43144–43153.

[121] Dixon, M. P., Pau, R. N., Howlett, G. J., Dunstan, D. E., Sawyer, W. H., and Davidson, B. E. (2002) The central domain of *Escherichia coli* TyrR is responsible for hexamerization associated with tyrosine-mediated repression of gene expression. *J. Biol. Chem.,* **277**, 23186–23192.

[122] Ray, W. K. and Larson, T. J. (2004) Application of AgaR repressor and dominant repressor variants for verification of a gene cluster involved in N-acetylgalactosamine metabolism in *Escherichia coli* K-12. *Mol. Microbiol.,* **51**, 813–826.

[123] Garces, F., Fernández, F. J., Gómez, A. M., Pérez-Luque, R., Campos, E., Prohens, R., Aguilar, J., Baldomà, L., Coll, M., Badía, J., and Vega, M. C. (2008) Quaternary structural transitions in the DeoR-type repressor UlaR control transcriptional readout from the L-ascorbate utilization regulon in *Escherichia coli*. *Biochemistry,* **47**, 11424–11433.

[124] Larson, T., Ye, S., Weissenborn, D., Hoffmann, H., and Schweizer, H. (1987) Purification and characterization of the repressor for the sn-glycerol 3-phosphate regulon of *Escherichia coli* K12. *J. Biol. Chem.,* **262**, 15869–15874.

[125] Mortensen, L., Dandanell, G., and Hammer, K. (1989) Purification and characterization of the *deoR* repressor of *Escherichia coli*. *EMBO J.,* **8**, 325–331.

[126] Jaeger, T. and Mayer, C. (2008) The transcriptional factors MurR and catabolite activator protein regulate N-acetylmuramic acid catabolism in *Escherichia coli*. *J. Bacteriol.,* **190**, 6598–6608.

[127] Iwig, J. S., Leitch, S., Herbst, R. W., Maroney, M. J., and Chivers, P. T. (2008) Ni(II) and Co(II) sensing by *Escherichia coli* RcnR. *J. Am. Chem. Soc.,* **130**, 7592–7606.

[128] Rohankhedkar, M. S., Mulrooney, S. B., Wedemeyer, W. J., and Hausinger, R. P. (2006) The AidB component of the *Escherichia coli* adaptive response to alkylating agents is a flavin-containing, DNA-binding protein. *J. Bacteriol.,* **188**, 223–230.

[129] Schreiter, E. R., Sintchak, M. D., Guo, Y., Chivers, P. T., Sauer, R. T., and Drennan, C. L. (2003) Crystal structure of the nickel-responsive transcription factor NikR. *Nat. Struct. Biol.,* **10**, 794–799.

[130] Phillips, C. M., Schreiter, E. R., Stultz, C. M., and Drennan, C. L. (2010) Structural basis of low-affinity nickel binding to the nickel-responsive transcription factor NikR from *Escherichia coli*. *Biochemistry,* **49**, 7830–7838.

[131] Xue, T., Zhao, L., Sun, H., Zhou, X., and Sun, B. (2009) LsrR-binding site recognition and regulatory characteristics in *Escherichia coli* AI-2 quorum sensing. *Cell Res.,* **19**, 1258–1268.

[132] Wang, S., Fleming, R. T., Westbrook, E. M., Matsumura, P., and McKay, D. B. (2006) Structure of the *Escherichia coli* FlhDC complex, a prokaryotic heteromeric regulator of transcription. *J. Mol. Biol.,* **355**, 798–808.

[133] Van Duyne, G. D., Ghosh, G., Maas, W. K., and Sigler, P. B. (1996) Structure of the oligomerization and L-arginine binding domain of the arginine repressor of *Escherichia coli*. *J. Mol. Biol.,* **256**, 377–391.

[134] Sträter, N., Sherratt, D. J., and Colloms, S. D. (1999) X-ray structure of aminopeptidase A from *Escherichia coli* and a model for the nucleoprotein complex in Xer site-specific recombination. *EMBO J.,* **18**, 4513–4522.

[135] Wang, S., Wu, Y., and Outten, F. W. (2011) Fur and the novel regulator YqjI control transcription of the ferric reductase gene yqjH in *Escherichia coli*. *J. Bacteriol.,* **193**, 563–574.

[136] Grinberg, I., Shteinberg, T., Hassan, A. Q., Aharonowitz, Y., Borovok, I., and Cohen, G. (2009) Functional analysis of the *Streptomyces coelicolor* NrdR ATP-cone domain: role in nucleotide binding, oligomerization, and DNA interactions. *J. Bacteriol.,* **191**, 1169–1179.

[137] Thaw, P., Sedelnikova, S. E., Muranova, T., Wiese, S., Ayora, S., Alonso, J. C., Brinkman, A. B., Akerboom, J., van der Oost, J., and Rafferty, J. B. (2006) Structural insight into gene transcriptional regulation and effector binding by the Lrp/AsnC family. *Nucleic Acids. Res.,* **34**, 1439–1449.

[138] Schleif, R. (2010) AraC protein, regulation of the l-arabinose operon in *Escherichia coli*, and the light switch mechanism of AraC action. *FEMS Microbiol. Rev.,* **34**, 779–796.

[139] Ramos, J. L., Martínez-Bueno, M., Molina-Henares, A. J., Terán, W., Watanabe, K., Zhang, X., Gallegos, M. T., Brennan, R., and Tobes, R. (2005) The TetR family of transcriptional repressors. *Microbiol. Mol. Biol. Rev.,* **69**, 326–356.

[140] Rigali, S., Derouaux, A., Giannotta, F., and Dusart, J. (2002) Subdivision of the helix-turn-helix GntR family of bacterial regulators in the FadR, HutC, MocR, and YtrA subfamilies. *J. Biol. Chem.,* **277**, 12507–12515.

[141] Chakerian, A. E., Tesmer, V. M., Manly, S. P., Brackett, J. K., Lynch, M. J., Hoh, J. T., and Matthews, K. S. (1991) Evidence for leucine zipper motif in lactose repressor protein. *J. Biol. Chem.,* **266**, 1371–1374.

[142] De Carlo, S., Chen, B., Hoover, T. R., Kondrashkina, E., Nogales, E., and Nixon, B. T. (2006) The structural basis for regulated assembly and function of the transcriptional activator NtrC. *Genes Dev.,* **20**, 1485–1495.

[143] Amouyal, M., Mortensen, L., Buc, H., and Hammer, K. (1989) Single and double loop formation when deoR repressor binds to its natural operator sites. *Cell,* **58**, 545–551.

[144] Palacios, S. and Escalante-Semerena, J. C. (2004) 2-Methylcitrate-dependent activation of the propionate catabolic operon (prpBCDE) of *Salmonella enterica* by the PrpR protein. *Microbiology,* **150**, 3877–3887.

[145] Schreiber, V. and Richet, E. (1999) Self-association of the *Escherichia coli* transcription activator MalT in the presence of maltotriose and ATP. *J. Biol. Chem.,* **274**, 33220–33226.

[146] Leonhartsberger, S., Ehrenreich, A., and Böck, A. (2000) Analysis of the domain structure and the DNA binding site of the transcriptional activator FhlA. *Eur. J. Biochem.,* **267**, 3672–3684.

[147] Sallai, L. and Tucker, P. A. (2005) Crystal structure of the central and C-terminal domain of the sigma(54)-activator ZraR. *J. Struct. Biol.,* **151**, 160–170.

[148] Tucker, N. P., Ghosh, T., Bush, M., Zhang, X., and Dixon, R. (2010) Essential roles of three enhancer sites in sigma54-dependent transcription by the nitric oxide sensing regulatory protein NorR. *Nucleic Acids. Res.,* **38**, 1182–1194.

[149] Schumacher, J., Zhang, X., Jones, S., Bordes, P., and Buck, M. (2004) ATP-dependent transcriptional activation by bacterial PspF AAA+protein. *J. Mol. Biol.,* **338**, 863–875.

[150] Klumpp, S. and Hwa, T. (2008) Growth-rate-dependent partitioning of RNA polymerases in bacteria. *Proc. Natl. Acad. Sci. USA,* **105**, 20245–20250.

[151] DeHaseth, P., Lohman, T., Burgess, R., and Record Jr, M. (1978) Nonspecific interactions of *Escherichia coli* RNA polymerase with native and denatured DNA: differences in the binding behavior of core and holoenzyme. *Biochemistry,* **17**, 1612–1622.
